# Flexible Antennas for a Sub-6 GHz 5G Band: A Comprehensive Review

**DOI:** 10.3390/s22197615

**Published:** 2022-10-08

**Authors:** Deepthi Mariam John, Shweta Vincent, Sameena Pathan, Pradeep Kumar, Tanweer Ali

**Affiliations:** 1 Department of Electronics and Communication Engineering, Manipal Institute of Technology, Manipal Academy of Higher Education, Manipal 576104, India; 2 Department of Mechatronics Engineering, Manipal Institute of Technology, Manipal Academy of Higher Education, Manipal 576104, India; 3 Department of Information and Communication Engineering, Manipal Institute of Technology, Manipal Academy of Higher Education, Manipal 576104, India; 4 Department of Electrical, Electronic and Computer Engineering, University of Kwazulu-Natal, Durban 4041, South Africa

**Keywords:** flexible antenna, sub-6 GHz, wireless body area network (WBAN), bending, specific absorption rate (SAR), multiple input multiple output (MIMO)

## Abstract

The ever-increasing demand and need for high-speed communication have generated intensive research in the field of fifth-generation (5G) technology. Sub-6 GHz 5G mid-band spectrum is the focus of the researchers due to its meritorious ease of deployment in the current scenario with the already existing infrastructure of the 4G-LTE system. The 5G technology finds applications in enormous fields that require high data rates, low latency, and stable radiation patterns. One of the major sectors that benefit from the outbreak of 5G is the field of flexible electronics. Devices that are compact need an antenna to be flexible, lightweight, conformal, and still have excellent performance characteristics. Flexible antennas used in wireless body area networks (WBANs) need to be highly conformal to be bent according to the different curvatures of the human body at different body parts. The specific absorption rate (SAR) must be at a permissible level for such an antenna to be suited for WBAN applications. This paper gives a comprehensive review of the current state of the art flexible antennas in a sub-6 GHz 5G band. Furthermore, this paper gives a key insight into the materials for a flexible antenna, the parameters considered for the design of a flexible antenna for 5G, the challenges for the design, and the implementation of a flexible antenna for 5G.

## 1. Introduction

Wireless communication has witnessed a huge transformation over the past few decades. The recent advancements in wireless communication need a huge number of devices connected to the internet which cannot be managed by the existing 4G LTE (fourth generation long term-evolution) system [1]. The fifth-generation (5G) communication system is a promising technology that fulfills the increasing requirements of data rates and also enables its integration with various services [2]. 5G can solve the issues that existed in the previous generation, in terms of bandwidth, QoS (quality of service), latency rate, and data transmission rates. The connection of a huge number of devices with high data rates, a low latency, more connectivity, and efficiency is achieved with 5G.

The 5G network will be beneficial to many industries, including improved health care, smart cities, 3-D imaging, the IoT (internet of things), and more. The entire spectrum of 5G is classified into mainly three frequency bands viz a low band ranging from 600 to 700 MHz, a sub-6 GHz band ranging from 3 to 5 GHz, and a millimeter-wave band ranging from 24 to 100 GHz [3,4,5,6]. Table 1 gives an overview of the 5G sub-6 GHz mid-band spectrum utilized in different countries [7]. A sub-6 GHz (Mid band) has become the main focus of researchers due to the limitation of mm-waves. Since mm-waves are high-frequency waves, they can only cover very short ranges. So, the implementation of mm-wave devices is expensive. Moreover, the implementation of sub 6-GHz could be achieved with the already existing 4G LTE bands thus making it an appropriate choice for 5G communication.

**Figure 1:**
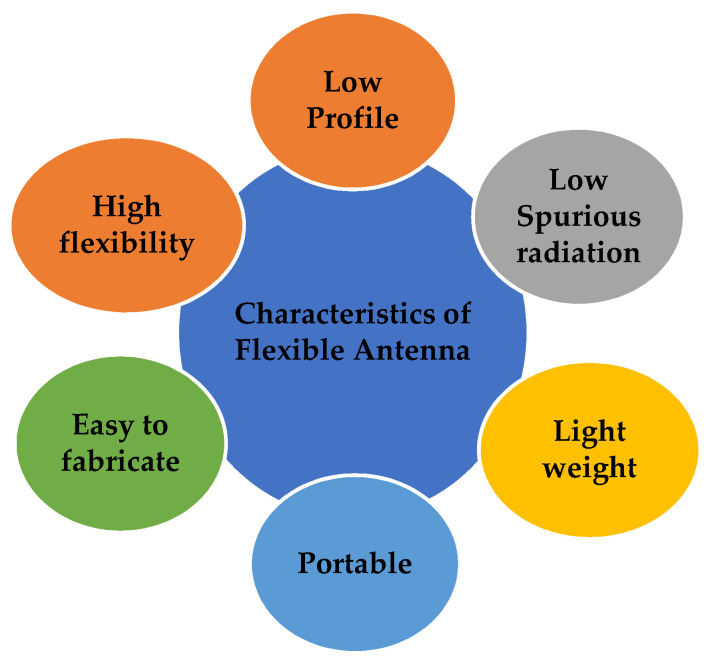
Characteristics of a flexible antenna.

One of the technological advancements with the outbreak of the fifth generation is in the area of flexible industry. Flexible devices are adaptable, lightweight, inexpensive, and environmentally friendly. Such flexible devices need an antenna to operate in particular frequency bands in order to give a good wireless connectivity. The general characteristics of a flexible antenna are given in Figure 1.

The market for flexible electronics is increasing due to the high demand for wearable devices, especially health monitoring systems. Therefore research on several flexible antennas has increased in recent years in biomedical applications [8,9].Flexible and conformal antennas provide good mechanical properties that make them suitable for wireless body area networks (WBANs) since continuous bending of the antenna should not degrade the performance of the antenna [10,11]. Moreover, the human body also has different dimensions and curvatures in different parts of the body. As a result, a flexible antenna plays a key role in this regard, as it must bend to match the curve of the human body when positioned in various places of the body [12].

**Figure 2:**
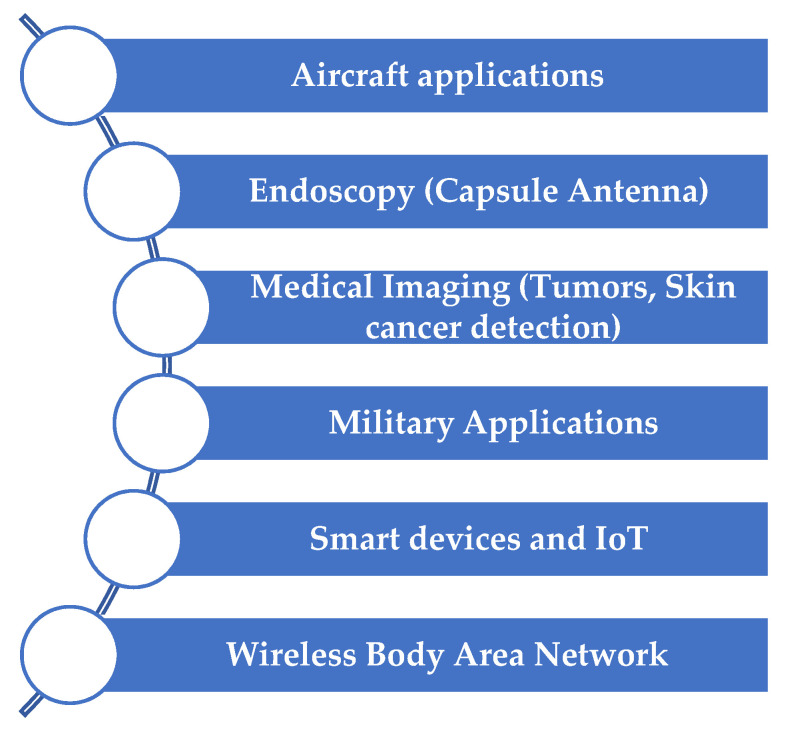
Applications of a flexible antenna.

Recently, the flexible antenna has marked a significant role in military applications. An all-textile cavity-backed substrate integrated waveguide (SIW) antenna is proposed for military applications in [13]. The main applications of flexible antennas are given in Figure 2.

Materials with good electrical and mechanical properties, as well as a low relative permittivity and loss tangent values are needed for the design of a flexible antenna [14]. Another requirement of these antennas is that they should operate with minimum deterioration in the proximity of the human body. The field of wearable antennas is challenging concerning fabrication. The space constraint while placed on specific body positions, the effects of the host body and the performance deterioration due to various structural deformations need to be considered while designing such wearable antennas [15].

In this paper, a detailed review of flexible antennas for the sub-6 GHz 5G band is reviewed. The main contribution of this article is as follows:

- Materials and design parameters needed for the design of flexible antennas are reviewed.
- A detailed classification of a single element, array, and MIMO flexible antennas in the recent literature is studied and a detailed analysis of different methodologies and their effect on the flexible antenna performance characteristics, such as reflection coefficient, gain, radiation pattern, the effect due to bending and the SAR are reviewed.
- Based on that, single element flexible antennas for sub-6 GHz 5G are reviewed by classifying the same into slot/slit-based, DGS-based, metamaterial-based, DRA-based, and transparent flexible antennas.
- Flexible array antennas for the abovesaid band are classified into slot/slit based, metamaterial based and transparent flexible antennas.
- The current state of the art in MIMO flexible antennas for the sub-6 GHz 5G band is reviewed with the classification of the slot/slit based, DGS-based, metamaterial-based, DRA-based, and transparent flexible MIMO antennas.
- Further, several design challenges and possible solutions for the implementation of flexible antennas are discussed.

The entire paper is organized as follows: Section 1 gives the introduction and theoretical concepts of the flexible antenna and the 5G technology. The materials for the design of flexible antennas are elaborated upon in Section 2. Parameters to be considered in the flexible antenna for sub-6 GHz 5G are presented in Section 3. Flexible antennas for a sub-6 GHz 5G band are reviewed in detail in Section 4. Challenges in designing a flexible antenna in sub-6 GHz 5G are given in Section 5, followed by discussions and conclusion in Section 6 and Section 7, respectively.

## 2. Materials for a Flexible Antenna

Flexible antennas are made of two kinds of materials: conductive materials and substrates. Both conductive materials and substrates need to be chosen in such a way that the antenna fabricated using these materials should show good radiation characteristics. In the design of any flexible antenna, the choice of substrate material is very important. The substrate is selected based on its dielectric characteristics, and capacity to bear mechanical deformations including bending, crumpling, and mechanical stresses. Conductive materials, on the other hand, are chosen in terms of their good electrical conductivity, resistivity, tensile strength, and ability to integrate with flexible substrates [16].

### 2.1. Conductive Materials

The choice of conductive materials for flexible antennas involves materials with a good electrical conductivity that provides high gain, bandwidth, and efficiency. Moreover, good conductive material will have resistance to degradation concerning mechanical deformation. The stiff architecture of the wearable antennas, which are created using a variety of conductive materials, limits their flexibility. Several smart textiles have been used for flexible antennas which provide a high level of flexibility. However, stretching and crumpling of the antenna will lead to the deterioration in the antenna’s performance [15].

Wearable antennas that are durable and frequency-tunable are created using conductive fibers which are embedded in a polymer substrate. However, this type of antenna is vulnerable to stretch because of the deformation effects in the flexible materials [17]. To overcome this, highly stretchable materials have come to the frontline such that deformation due to the high stretchability of the antenna can be well managed.

**Figure 3:**
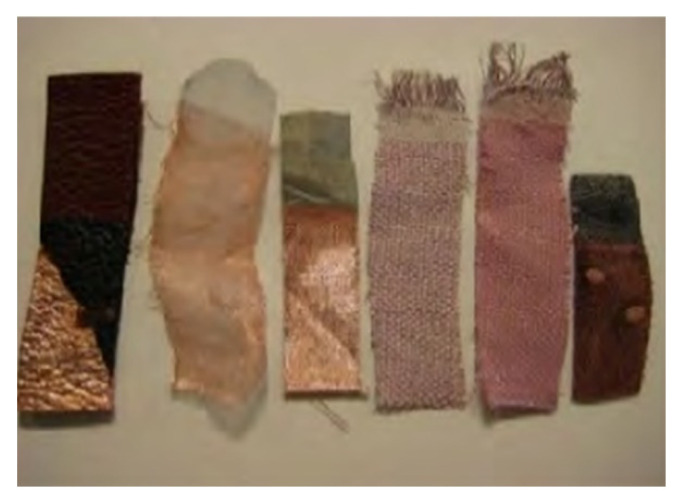
Different textile conductive materials for flexible antennas. Reprinted from ref. [15].

Stretchable, doped materials, such as silver-integrated rubber [18], stretchable fabric [19], polymer composite (MXene ink) [20], etc. are integrated for wearable applications. Several new materials are used to increment the performance of stretchable materials, including indium, meshed wires, and transparent fabrics. A new Ti3C2Tx/PVDF meta composite with a negative permittivity has gained greater attention in recent days due to its unique properties and thus finds applications in the field of the flexible antenna [21]. Several conductive textile materials for flexible antennas are shown in Figure 3.

Applications such as wireless body area networks, telemetry, etc., utilize conductive materials to sustain repetitive pressure when continuously bent [22]. Therefore, these materials should be selected in such a way that they will not degrade the antenna’s performance.

The high electrical conductivity of the nanoparticles makes them a suitable choice for the fabrication of flexible antennas. Silver and copper-based nanoparticles are commonly used. Silver nanoparticles exhibit a good electrical conductivity, adhesion, and visibility [23]. A carbon nanoparticle integrated with polyaniline (PANI) shows good mechanical and electrical properties and silver nano wire (AgNW) shows a good conformality [24].

Copper-based conducting materials such as copper mesh are also seen in the literature for the fabrication of flexible antennas due to their good electrical properties, easy availability, and good radiation properties [25]. Nickel-embedded metallic mesh is used as a conductive electrode which shows a good mechanical stability and a good electrical conductivity than the traditional metallic mesh [26]. Meshed fabric and nanoflakes are also used as the conductive materials for the design of flexible antenna due to their good mechanical and thermal stabilities [27,28].

Transparent conductors also have come into the frontline along with the transparent substrates for the design of flexible antennas. Some transparent conductors that are reported in the literature are indium-tin oxide, silver coated polyester film (AgHT), aluminum zinc oxide, etc. However, such conductors exhibit a low conductivity even though they provide a good visual clutter [29,30]. Two dimensional materials such as MXenes and graphene have come into the frontline especially for communication devices that need a high electrical conductivity. MXenes possess a high electrical conductivity and graphene has a good chemical stability. In [31], the conductivity of the graphene assembled film (GAF) is improved by applying a high temperature annealing process. Graphene-based conductors with larger sizes and areas increase the electrical conductivity of the conducting material [32].

Table 2 shows the various conductive materials utilized for the design of flexible antennas and their characteristics [27,28].

### 2.2. Substrate Materials

Substrate materials play a crucial role in the design of any flexible antenna. The substrate used in a flexible antenna should have a low relative permittivity, a low loss tangent value, and a high thermal conductivity. Since flexible electronics have gained greater application in body area networks, the choice of the flexible substrate should be in such a way that it should be very conformal to maintain the deformation criteria, such as bending, stretching, etc.

Various substrate materials are seen in the literature for the development of flexible antennas, as shown in Table 3. Paper, fabrics, plastic, and polymer substrates are commonly used in the development of flexible antennas. Paper has many advantages to be used as a flexible substrate since it is cost-effective and easy to manufacture. Paper substrates are low profile and lightweight and thus make them easier to be integrated into flexible devices. One of the main disadvantages of paper, as a substrate for the design of flexible electronics, is that when given continuous bending the paper becomes very fragile, thus degrading the antenna performance completely. Another disadvantage of the paper substrate is its high moisture absorption which in turn affects the antenna performance [33].

Textile or fabric substrates are seen in the literature for the design of flexible antennas. Fabrics are low in profile and can be easily integrated with the conducting materials, making them feasible to be used in wearable applications. However, fabrics have certain disadvantages, such as a high level of moisture absorption, and they are also prone to cracks after many bending cycles. This turns out to be a notable disadvantage while used in medical applications that involve the repeated bending of antennas around different radii [34]. In general, fabric materials possess an extremely low dielectric constant that further reduces the surface wave losses and improves the impedance bandwidth of the antenna. The measured dielectric values of the different textile fabrics are provided in [35].

The polymer-based substrate is used in the field of wearable electronics due to its stretchability, flexibility, and robustness [24]. Polyethylene terephthalate (PET) and polyethylene naphthalate (PEN) are utilized in several flexible antennas due to their outstanding mechanical properties as well as their resistance to moisture absorption [29,36]. Liquid crystal polymer (LCP), which is a thin film substrate, is utilized in the development of a flexible antenna due to its very low loss tangent, low dielectric constant, and low thermal expansion characteristics. The low thickness of the LCP substrate reduces the antenna volume and also enables it to work in a bending state [37].

In [38], a flexible PIFA antenna is designed using a PEN substrate for 5G applications. A circularly polarized wearable antenna that operates at 5.8 GHz is proposed on a polydimethylsiloxane (PDMS) substrate [39]. Highly conformal characteristics, lightweight, and a good mechanical stability of the substrate make it a good choice for a wearable antenna design. The comparison of various flexible materials and their properties are given in Table 3 [27].

The properties of any substrate are characterized by the permittivity and the loss tangent. The permittivity of the substrate is given in Equation (1) [40].

(1) $$\;\varepsilon =\varepsilon_{o}\varepsilon_{r}$$

where $\varepsilon_{r}$ denotes the substrate’s relative permittivity, $\varepsilon_{o}$ is the free space’s permittivity. The relative permittivity of the substrate can be represented in Equation (2) [40].

(2) $$\varepsilon_{r}=\varepsilon_{r}^{r}+j\varepsilon_{r}^{i}$$

where $\varepsilon_{r}^{r}$ is the real part of the relative permittivity which is popularly known as the substrate’s dielectric constant and $j\varepsilon_{r}^{i}\;$is the imaginary part of the relative permittivity.

The loss tangent (tanδ) of the dielectric material is as in Equation (3) [40].

(3) $$tan\delta =\frac{\varepsilon_{r\;}^{i}}{\varepsilon_{r}^{r}}$$

The quality (Q) factor of the flexible antenna depends upon the dielectric constant of the substrate material. The choice of materials with a low dielectric constant decreases the quality factor and thereby increases the bandwidth. The resonant frequency of the antenna is also related to its dielectric constant. The choice of the substrate with a low dielectric constant can increase the antenna’s resonant frequency and vice versa [40]. Thus, the choice of the flexible substrate with a low loss tangent and low permittivity is the best in the design of flexible antennas. Table 4 shows the various substrate materials used in the design of flexible antennas with their advantages and disadvantages.

## 3. Parameters to Be Considered for the Design and Analysis of a Flexible Antenna

The design and analysis of the flexible antenna need a wide range of parameters to be considered, rather than the conventional rigid antenna counterparts. Apart from the design considerations of choosing proper conductive and substrate materials for the flexible antenna as discussed in Session 2, certain parameters must be emphasized for the design, as well as the analysis of the flexible antenna which are discussed as follows:

### 3.1. Reflection Coefficient

One of the main parameters for the design of a flexible antenna is the reflection coefficient. The ratio of the amplitude of the reflected wave to the incident wave is known as an antenna’s reflection coefficient. The reflection coefficient ($S_{11}$) represents how much power is reflected from the antenna. The acceptable value of $S_{11}$ is −10 dB so that if the antenna is designed with a low loss, most of the power given to the antenna is radiated.

The resonance bandwidth and resonant frequency of the antenna can be used to describe the radiation properties of the flexible antenna. The resonance frequency range at the specified return loss of −10 dB is known as the antenna’s resonant bandwidth. Therefore, the reflection coefficient is a parameter that must be considered for the design of a flexible antenna so that any shift in frequencies due to bending, crumpling, temperature difference, or interaction with the human body, should not affect the considerable return loss of the flexible antenna.

### 3.2. Gain

Another figure of merit that describes the performance of a flexible antenna is the gain. The antenna must have an acceptable gain for the intended use to be accepted in the wearable market. A flexible antenna should exhibit a positive gain to become more efficient for wearable applications. An average gain of more than 3 dBi is needed for the flexible antenna for the smooth working in 5G applications.

Incorporating array, as well as MIMO structures, can improve the gain of the antenna. A greater patch volume can also increase the gain, but an increase in the antenna volume would not be a good choice for wearable applications where the antenna has to be conformal and should accommodate in less space.

### 3.3. Radiation Pattern and Radiation Efficiency

A flexible antenna for 5G needs a directional radiation pattern for applications such as cellular networks, base stations, global positioning systems (GPSs) etc., so that the radiation of the antenna becomes more concentrated in only some directions than in others. Applications, including wireless body area networks incorporate antennas with directional radiation patterns, such that the radiation should be focused on a particular direction. However, an omnidirectional radiation pattern is required in some specific flexible devices incorporating technologies, including Bluetooth, Wi-Fi, etc. Furthermore, another important factor that contributes to the performance of the flexible antenna is the radiation efficiency. It is the metrics that describe how efficiently the antenna transmits and receives the radio frequency signals. The radiation efficiency of the flexible antenna should be at acceptable level for the smooth working of a flexible antenna in 5G applications.

### 3.4. Bending Analysis

Flexibility, as well as bending tests for on-body measurements, need to be performed in realistic environments. The mechanical deterioration that includes bending, stretching, etc., degrades the performance of the antenna by shifting the frequency of resonance, a change in the gain and radiation patterns, etc., of the antenna for any specific application.

Moreover, the application of a flexible antenna in a wireless body area network involves continuous bending of the antenna across different curvatures of the body. The human body exhibits different curvatures at different positions of the body [41]. Therefore, the positioning of the antenna across such different positions shows different antenna characteristics.

The bending analysis of the flexible antenna across different radii shifts the resonant frequency of the antenna. This is because when the antenna is bent, it varies the effective length of the antenna and thus varies the resonant frequency.

**Figure 4:**
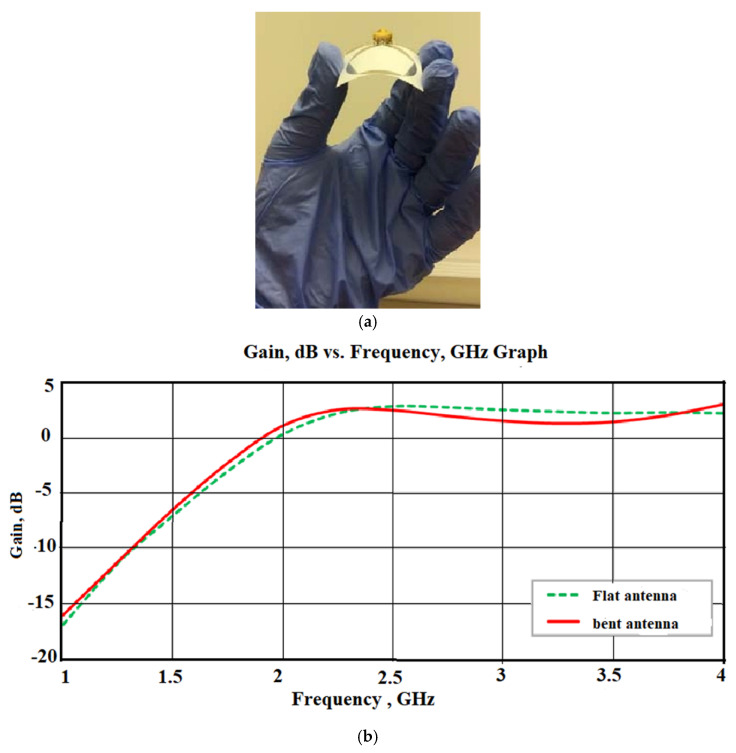
Effect of bending on the gain: (**a**) Bending of a slotted dipole antenna; (**b**) Gain analysis of the antenna under flat and bent conditions. Reprinted from ref. [42].

The bending of the flexible antenna also changes the gain of the antenna which affects its performance. It is observed that as we increase the radius of the curved surface, the gain and antenna performance increase and on further increasing the radii, it decreases. However, for the surface-like arm, which is similar to a planar surface, the performance is optimum. Therefore, while increasing the bending radius, the antenna gain increases since the antenna’s effective areas are more and thereby contribute a good gain. The bending of the conformal antenna should therefore be optimized in such a way that the antenna performance, such as the resonant frequency, gain, etc., should have a very negligible impact on the antenna performance. Figure 4 shows the effect of bending on the gain for a slotted dipole antenna under flat and bend conditions [42].

### 3.5. Specific Absorption Rate (SAR)

The absorption of electromagnetic radiation with the use of wearable devices, including antennas, is governed by the specific absorption rate (SAR) limits. The amount of electromagnetic radiation absorbed per unit mass of the human body is known as the specific absorption rate.

As far as a WBAN is considered, to guarantee its safety it must be ensured that the SAR value must be as per the recommended value. The highest permissible limit of the SAR for 1 gm of tissue, according to US guidelines, is 1.6 W/kg and the SAR maximum value for any 10 g of tissue has been set at 2 W/kg by the International Commission on Non-Ionizing Radiation Protection (ICNIRP) for Europe [43].

**Figure 5:**
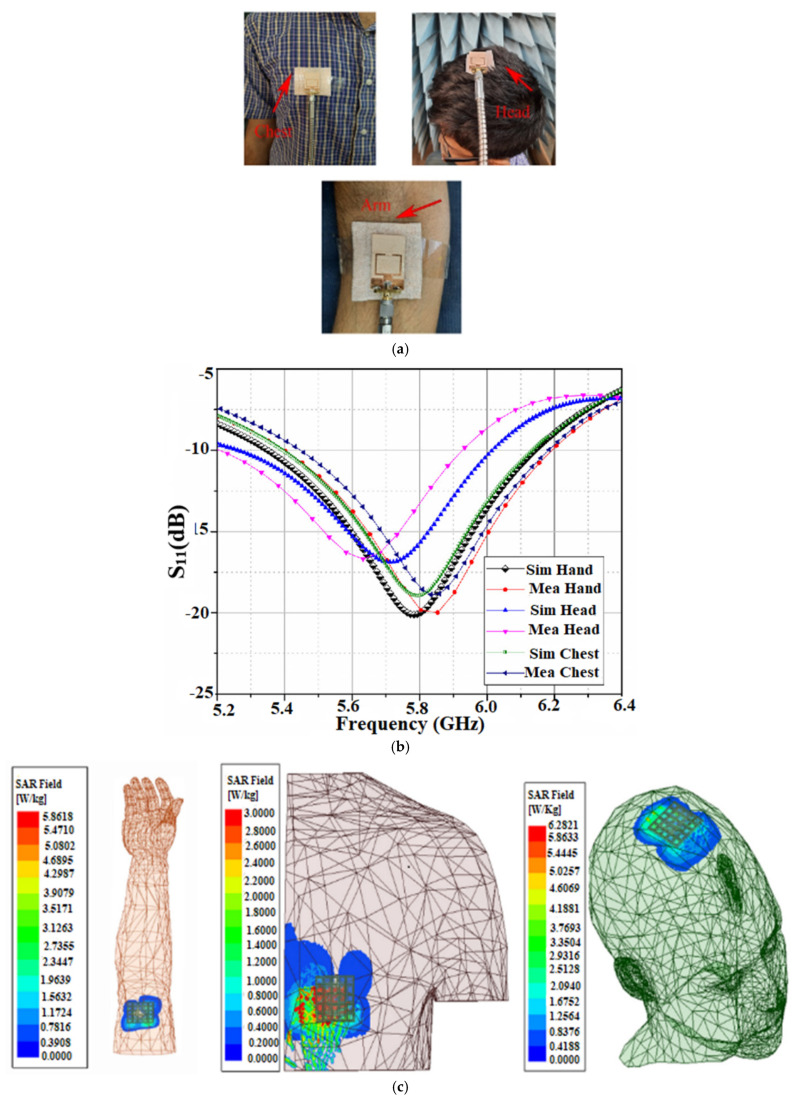
Relevance of the SAR on the design of a flexible antenna: (**a**) AMC-backed antenna on various parts of the body; (**b**) Reflection coefficient behavior on the human body; (**c**) SAR of the antenna on different parts of the human body—hand, chest, and head. Reprinted from ref. [44].

Therefore, one of the main considerations in the use of a wearable antenna is to limit its SAR following international standards. An AMC-backed (artificial magnetic conductor) antenna on different parts of the human body is proposed in [44] for a ISM 5.8 GHz band, as shown in Figure 5a–c show the reflection coefficient behavior and the SAR distribution rate of the antenna on the various parts of the human body, respectively [44].

To analyze the SAR values of the different body parts at a particular frequency, the dielectric properties, such as the dielectric constant and conductivity of the different tissues, should be considered. Moreover, the SAR values differ depending upon the body part where the antenna is placed, and the thickness of the tissues associated with the specific body part. Table 5 depicts the various tissue layers and their thickness in various parts of the body [43].

When the antenna is placed close to the human body, its performance suffers because of the lossy human body tissue interactions. When placed near the human body, having a high conductivity and dielectric constant, the wearable antenna shifts the frequency of resonance. The dielectric constant and conductivity of the muscle tissue are related to the resonant frequency. The dielectric constant of the tissue is inversely proportional to the resonance frequency and directly proportional to the conductivity of the tissue [44]. Moreover, with the variation in body parts, the conductivity of the tissue is varied, and with the variation in type (skin, fat, muscle, etc.), the permittivity of the human tissue also varies. The reflection coefficient is affected by the difference in the permittivity and conductivity levels, which in turn affects the antenna’s performance. The dielectric characteristics of different tissue layers for different frequencies are shown in Table 6 [45].

It is to be ensured that the SAR should be within the permissible limit to be used for wearable antenna applications. The placement of the antenna in various human body parts having different dielectric constant values, in turn, affects the antenna performance. Hence, these considerations should be considered while designing a wearable antenna.

## 4. Flexible Antennas for a Sub-6 GHz 5G Band

The transition to 5G is anticipated to make the best use of the sub-6 GHz band, necessitating a widespread deployment of devices with adequate network coverage. Devices that work on high data rates need an antenna to be wideband to integrate multiple applications. The increasing demand for smart devices needs an antenna to be compact and capable of mounting on any irregular surface. These antennas should also maintain a good performance concerning several parameters such as gain, impedance bandwidth, radiation efficiency, and radiation pattern. The transformation of the antenna from a non-flexible to flexible antenna needs the research of various antenna structures and flexible substrates. The flexible antenna for the recently released sub-6 GHz 5G band thus needs a detailed discussion.

**Figure 6:**
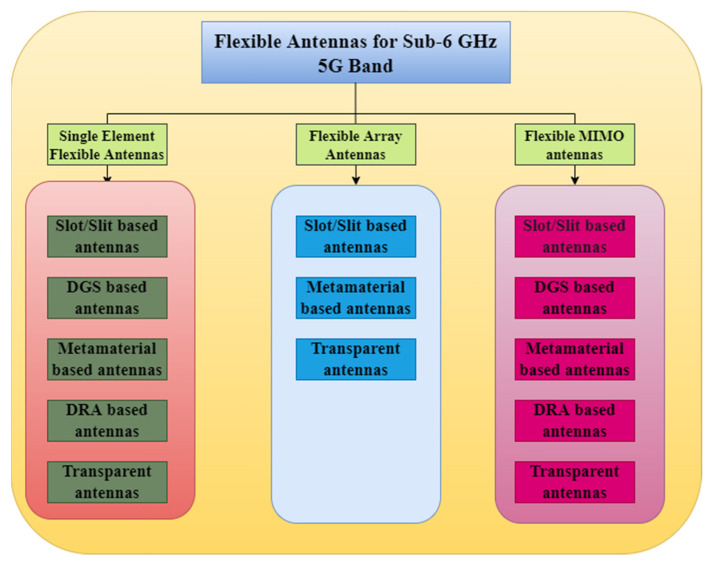
Overview of flexible antennas for the sub-6 GHz 5G band.

The following section gives an overview of flexible antennas for the mid-band 5G which includes the single element flexible antenna, the flexible array antenna, and the flexible MIMO antenna. A single element antenna is reviewed with a classification of slot/slit-based, DGS-based, metamaterial-based, DRA-based, and transparent flexible antennas. A flexible array antenna is reviewed on the classification as slot/slit based, metamaterial-based, and transparent flexible antennas. Flexible MIMO antennas are reviewed, by classifying them into slot/slit-based, DGS-based, metamaterial-based, DRA-based, and transparent flexible antennas. The overview of different flexible antennas for the sub-6 GHz 5G band is shown in Figure 6.

### 4.1. Single Element Flexible Antennas for the Sub-6 GHz 5G Band

#### 4.1.1. Slot/Slit Based Flexible Antenna

Slot elements are utilized to increase the impedance bandwidth of the antenna using the coupling mechanism in the radiating patch, as well as the ground plane. High gain, efficiency, a larger bandwidth, and a high mutual coupling value can be obtained with the slot antenna [46]. The following section provides slot/slit-based flexible antennas suitable for sub-6 GHz 5G applications.

**Figure 7:**
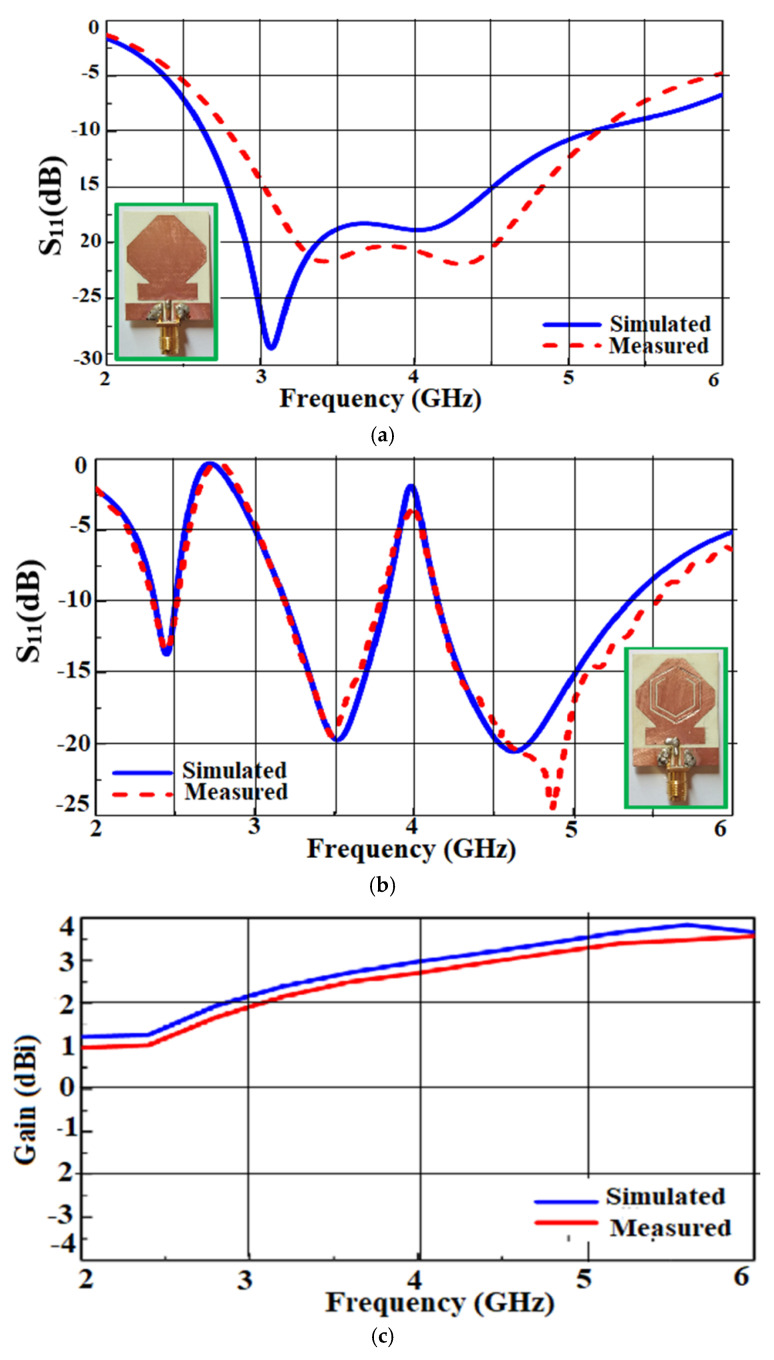

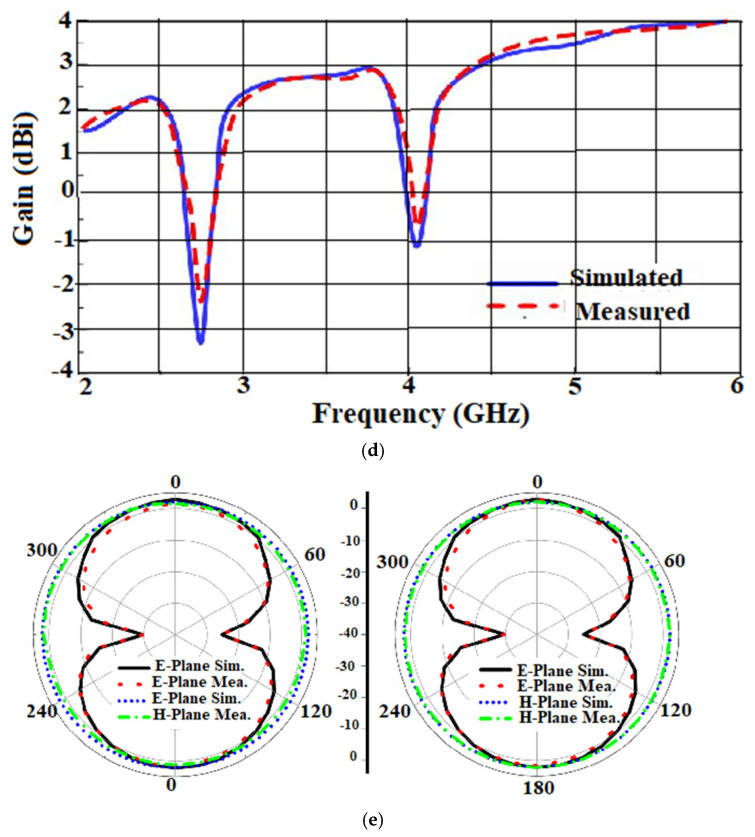
Multiband slotted antenna: (**a**) Return of the loss of the wideband antenna; (**b**) Return of the loss of the triband antenna; (**c**) Gain analysis of the wideband antenna; (**d**) Gain analysis of the triband antenna; (**e**) Comparison of the radiation patterns of the antenna at 3.5 GHz. Reprinted from ref. [47].

In [47], a wideband and tri-band antenna that can be utilized for 5G sub-6 GHz communication with independently controllable notch bands, is proposed. The wideband monopole antenna consists of an octagonal geometry with truncated edges that improves the impedance bandwidth of the antenna. The bandwidth is increased by a rectangular stub covering the whole sub-6 GHz band. The slots are introduced in the wideband antenna structure to achieve the band notches to mitigate the C-band and Wi-Max band. The wideband antenna shows a frequency range of 2.8 to 5.35 GHz, and the tri-band antenna is resonating at frequencies viz. 2.45 GHz, 3.5 GHz, and 4.7 GHz. Figure 7a,b shows the S-parameter of the wideband and tri-band antennas, respectively. A gain of 3.75 dBi is achieved all along the covered spectrum, as shown in Figure 7c,d.

A directional radiation pattern is achieved for the proposed antenna over the entire band, as shown in Figure 7e. The bending analysis is performed to check the conformability of the antenna. For this, the wideband and triband antenna is bent on a Styrofoam cylinder over various radii of 10, 25, and 40 mm. It is seen that there is not much effect on the return of the loss on the various bending radii.

**Figure 8:**
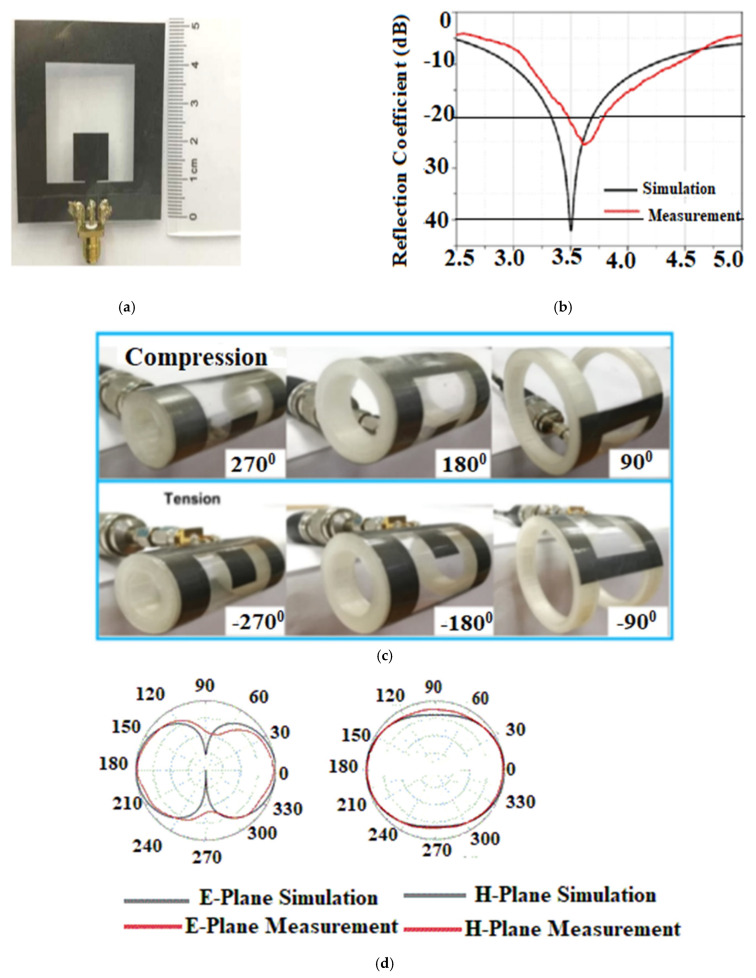
Conductive GAF slotted antenna sensor: (**a**) Fabricated prototype; (**b**) S-parameter of the antenna sensor; (**c**) Bending analysis set-up; (**d**) Radiation Pattern. Reprinted from ref. [48].

Another wideband antenna using conductive graphene film (GAF), is proposed in [48], implementing the slot in the radiating patch to achieve a bandwidth of 3.12–4.42 GHz, that covers the 5G band for the wireless wearable sensor application. The resonant frequency and impedance of the antenna sensor are achieved by using a short rectangular tuning stub. Figure 8a,b shows the antenna prototype and S-parameters, respectively. The bending analysis is carried out with the help of poly(lactic) acid or PLA rings of different testing radii, as shown in Figure 8c. The antenna is tested on an arm under 100 cycles of testing, involving flat and bent conditions. It is noted that the resonant frequency was shifted abruptly but is reverted to the original state when the arm is in a flat position. The antenna shows a directional radiation pattern, as shown in Figure 8d.

**Figure 9:**
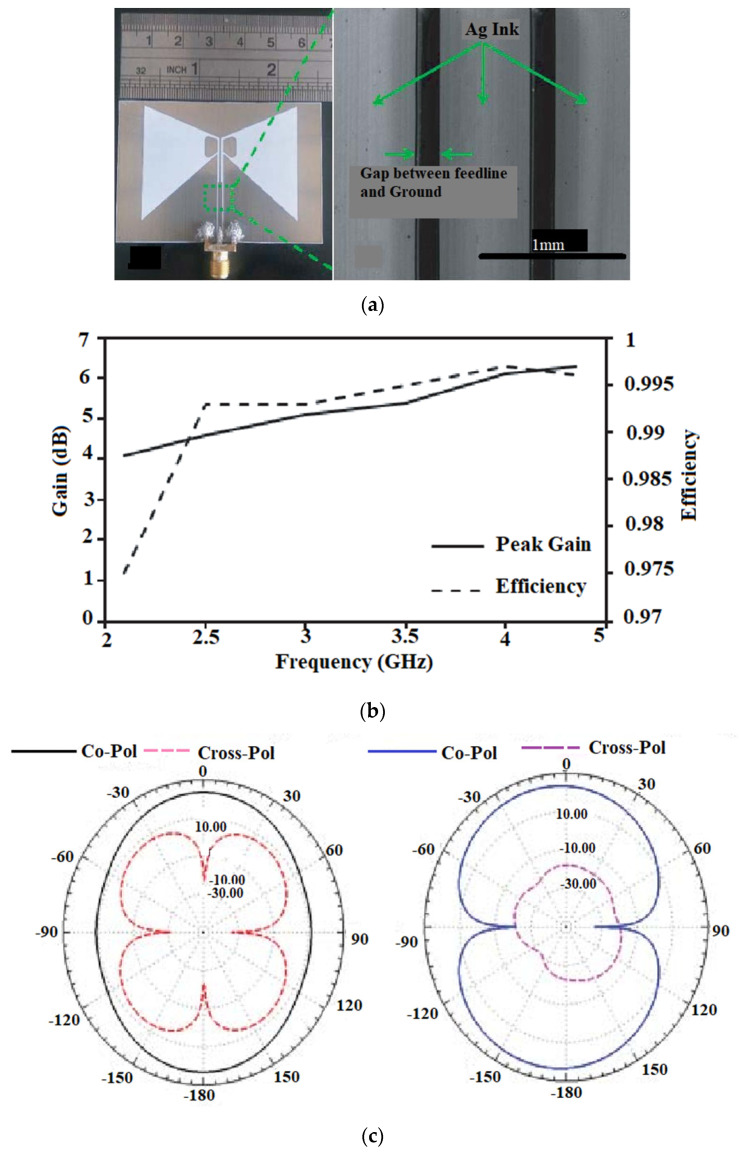

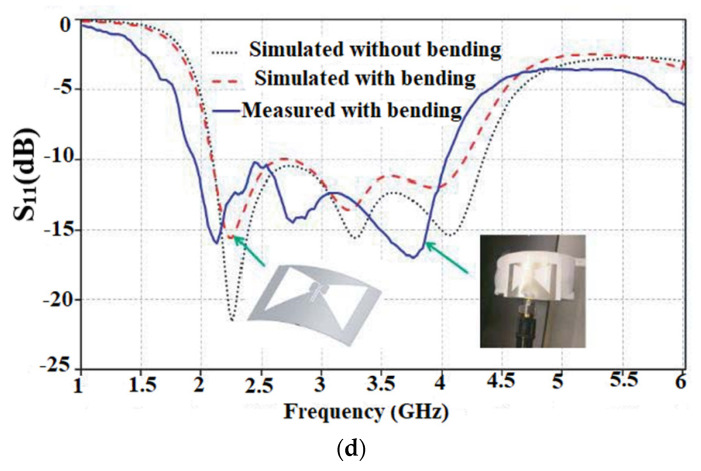
Bowtie slotted antenna: (**a**) Fabricated prototype; (**b**) Gain analysis; (**c**) Radiation Pattern; (**d**) Impact of the bending. Reprinted from ref. [49].

Another approach of the bowtie slot antenna for achieving a wideband with an improved gain, is proposed in [49], where the antenna is designed using a flexible PET substrate, as shown in Figure 9a. To obtain a high gain and a larger bandwidth, the design includes an asymmetric bowtie flare angle with a metal strip incorporated beside a bowtie slot. The antenna covers a bandwidth of 2.1–4.35 GHz with a peak gain of 6.3 dBi at 4.35 GHz as shown in Figure 9b.

The antenna exhibits an omnidirectional radiation pattern, as shown in Figure 9c. When the bending analysis of the antenna is carried out, it is observed that there is a variation in the frequency shift during the antenna testing phase, due to the increase in the dielectric constant value of the cylinder used for the bending analysis. This is due to the fact that with an increase in the dielectric constant, the resistive part of the input impedance reduces, and the reactance becomes capacitive. Figure 9d shows the impact of bending on the antenna return loss.

**Figure 10:**
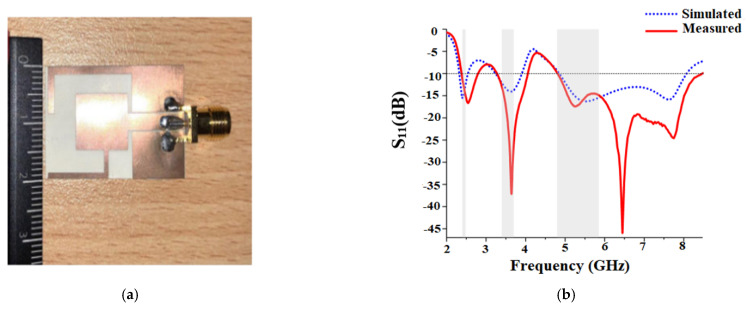

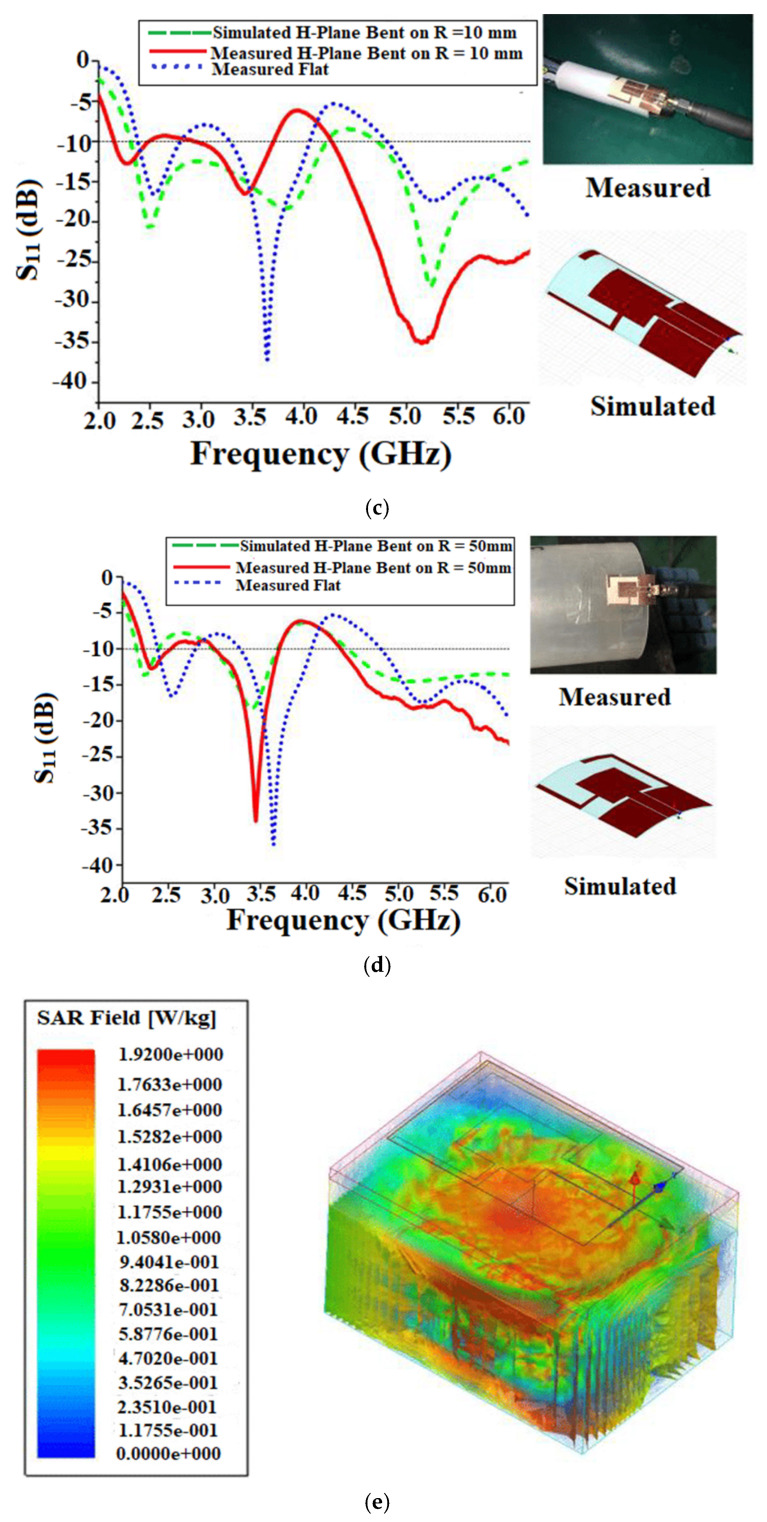
Multiband antenna on a LCP substrate: (**a**) Fabricated prototype; (**b**) S-parameter; (**c**) Measured and simulated S-parameter along the H-plane bent on the R = 10 mm; (**d**) Measured and simulated S-parameter along the H-plane bent on the R = 50 mm; (**e**) Simulated SAR value of 10 g of tissue on a human tissue model at 5 GHz. Reprinted from ref. [50].

In another approach in [50], a CPW antenna using an LCP substrate that combines two strips, one each on the radiating as well as the ground plane, is utilized for achieving a multiband operation, as shown in Figure 10a. The proposed antenna has three operating bandwidths 2.38–2.79 GHz, 3.27–4.05 GHz, and 4.80–8.44 GHz, respectively, as shown in Figure 10b. The average gain of 0.65 for 2.4 GHz, 2.26 for 3.5 GHz, and 2.6 dBi for 5.5 GHz are achieved. The antenna has a directional radiation pattern in both the E-plane and H-plane. The bending analysis of the antenna under the two radii (R = 10 mm and R = 50 mm), is shown in Figure 10c,d, respectively. It is observed that there is a slight variation in the $S_{11}$ of the antenna in all of the frequency bands. The SAR of the antenna is evaluated by placing the antenna at a distance of 5 mm from the model, which consists of several layers of tissue with different thicknesses. The maximum SAR values are found to be 0.93, 0.89, and 1.92 for 10 g tissue for frequencies of 2.4, 3.5, and 5 GHz, respectively, as shown in Figure 10e.

#### 4.1.2. DGS-Based Flexible Antenna

DGS is a structure where defects and slots are created in the ground plane of the antenna. These structures provide a wide bandwidth, a higher efficiency, and a low mutual coupling [46]. The following section provides the DGS-based flexible antennas suitable for sub-6 GHz 5G applications.

**Figure 11:**
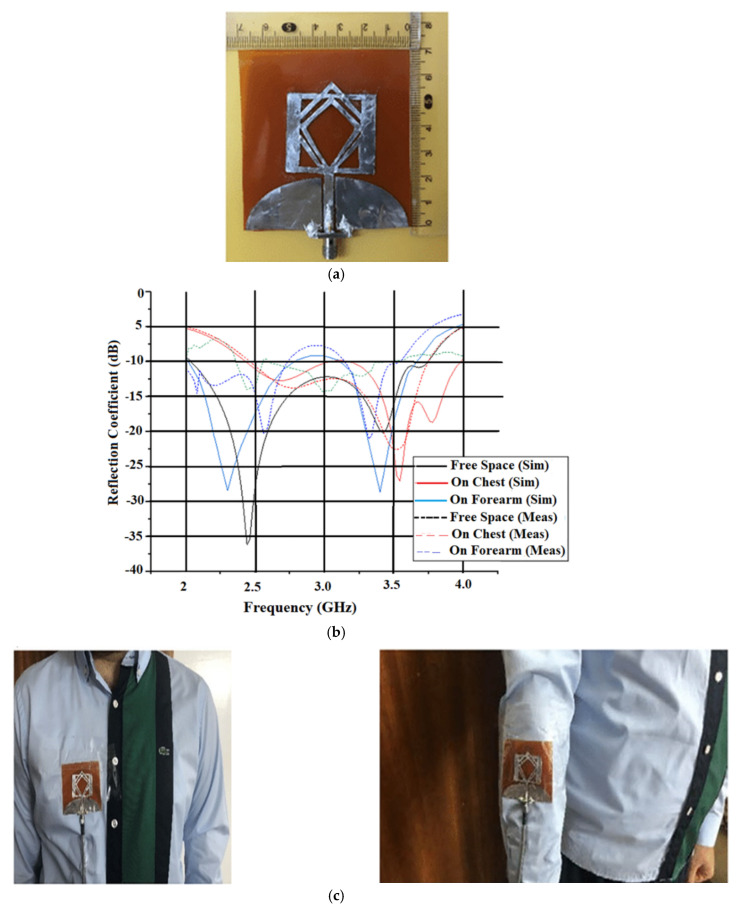

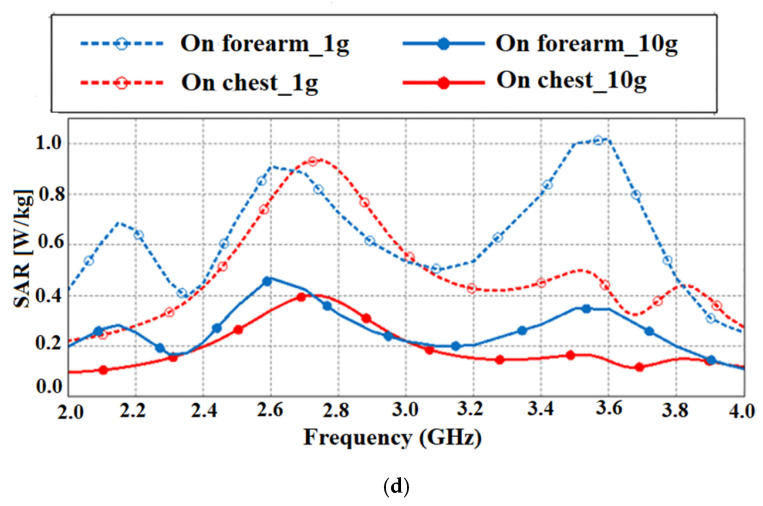
Monopole antenna with rhombic structures for the 5G: (**a**) Fabricated prototype; (**b**) Reflection coefficient of the antenna on the chest and forearm; (**c**) Proposed antenna on the chest (**left**) and forearm (**right**); (**d**) SAR analysis of the proposed antenna at a distance of d = 5 mm on the chest and forearm. Reprinted with permission from ref. [51]. Copyright 2020 John Wiley & Sons, Inc.

A highly efficient and enhanced bandwidth antenna is proposed in [51], where a monopole antenna operating in 2.45 GHz and 3.5 GHz is designed for WBAN/WLAN and 5G applications. The antenna that is fabricated on a flexible Kapton polyimide substrate is fed using a CPW feed. The antenna has two rhombic elements and a partial ground plane, as shown in Figure 11a. The S-parameter of the antenna is given in Figure 11b.

To validate the performance of the antenna and to ensure its safe usage for wearable applications, the SAR analysis of the antenna is carried out with the proposed antenna. The antenna is placed on the chest and forearm at a 5mm distance from the human body, as shown in Figure 11c. The maximum SAR value for 1 g of tissue is 0.908 and 0.782 in the forearm and chest, respectively, and for 10 g of tissue, it is 0.468 and 0.342 at 2.6 GHz, which is well below the international limits for safe SAR values, as shown in Figure 11d.

The DGS is also incorporated for gain enhancement. Such a flexible wideband antenna with an improved gain is proposed in [52]. The antenna volume consists of a CPW feedline, two L-shaped elements, a matching stub, and a DGS fabricated on a flexible Kapton polyimide substrate. A defected ground structure along with the L-shaped elements improved the bandwidth. Further adding of the matching stub helps in achieving a wideband from 1.77 to 6.95 GHz. A gain of 5.9 dBi and an efficiency of 90%, respectively, are achieved. The bending analysis of the antenna is performed for five bending radii 50, 20, ∞, −50, and −20 mm using simulation. It is seen that the bending across these two radii has not much difference in the performance of the antenna. The antenna shows a directional radiation pattern in the E-plane and an omnidirectional radiation pattern in the H plane. The radiation pattern is slightly affected across the bending around the radii of 20 and −20 mm. However, no null is seen in the broadside direction.

Ground planes can act as a shield to safeguard the users from harmful radiation. In [53], a wearable dual band ring-shaped folded antenna with a minimum SAR and a high gain is proposed. The antenna comprises two substrates, one which is rigid and acts as the main conductor, and the other which is a textile material that is a ground plane to protect the user from the backward radiation which reduces the SAR. The antenna could achieve a bandwidth of 5.7% (2.40–2.54 GHz) and 4.0% (3.38–3.52 GHz). The antenna has a gain ranging from 6.6 to 6.8 dB and 8.9 to 9.0 dB with an efficiency of 94% and 93% for the lower and higher frequency bands, respectively.

The antenna is tested for the bending analysis along a cylinder of the radius R at different radii in the x and y directions. It is seen that there is no shift in the frequency compared to the flat condition. To check the suitability of the antenna as a wearable application, the antenna is tested in free space and on a human body phantom. To ensure the safety for the user, the SAR is estimated for 1 g and 10 g of tissues for both frequencies. The SAR values are 0.2 and 0.1 W/kg for 1 g of tissue and 10 g of tissue, respectively, at 2.45 GHz and 0.1 and 0.04 W/kg for 1 g of tissue and 10 g of tissue at 3.45 GHz, respectively, which makes the antenna suitable for body area networks. The antenna shows a directional radiation pattern.

#### 4.1.3. Metamaterial-Based Flexible Antenna

Metamaterials (MTMs) are artificial structures that exhibit unique electromagnetic properties that cannot be seen in natural materials. These materials provide a low resonant frequency, compared to the resonant frequency of the antenna, resulting in the antenna miniaturization and also helping in enhancing the performance characteristics of the antenna including the gain, bandwidth, etc. The miniaturization of the antenna, based on MTMs, can be of various types, such as a metamaterial loading, a metamaterial inspired antenna, a meta surface loading, and a composite right/left hand material(CRLH) [54]. The following section gives a review of the metamaterial-based flexible antenna in the literature that is suitable for sub-6 GHz 5G applications.

A low-profile and a dual-band CPW-fed elliptical ring with a split-triangular patch antenna with an enhanced gain, is proposed in [55]. The antenna is designed on a flexible polyimide material and resonates at 2.60 GHz with a bandwidth ranging from 2.52–2.62 GHz and at 3.48 GHz with a bandwidth ranging from 3.31–3.64 GHz, observed at two frequency bands. The elliptical complementary split ring resonator (ECSRR) improved the gain of the antenna to 2.39 dBi and 1.75 dBi, respectively, for 2.60 GHz and 3.48 GHz. The triangular slot as well as the rectangular slit increased the electrical length for tuning and thus making the antenna work for the dual-band operation. To check the conformal nature of the antenna, a convex bending across the different radii of 30, 15, and 10 mm is performed and it is found that the performance of the antenna is not significantly changed with the bending effect. The radiation pattern of the antenna at 2.6 and 3.48 GHz gives a directive radiation pattern across the E-plane and an omnidirectional radiation pattern in the H-plane.

In another approach in [56], a circularly polarized metamaterial-loaded flexible antenna is fabricated on a flexible liquid crystal polymer substrate. Using metamaterial comprising of split ring resonators and a Complementary Split-ring Resonator (CSRR) on either side of the feedline and ground, helped in achieving a circular polarization. The antenna provides a bandwidth ranging from 3.4 to 3.78 GHz and 5.7 to 6.9 GHz. The boresight axial ratio is found to be less than 3 dB in both bands. The bending test of the antenna is carried out at different bending angles of 30°, 45°, 60°, and 120°. The impact of bending is mostly seen in the second band. The 30° angle shows a triple band variation. However, there is no significant variation for other bending angles compared to the planar antenna. The highest axial ratio is found at 30° further than the others.

An artificial magnetic conductor is another kind of meta surface loading that is seen for the miniaturization of the antenna, as well as improving the performance of the antenna. A wearable high gain antenna that operated at 3.5 GHz, as well as 5.8 GHz with a low SAR is proposed in [57]. The antenna, which is placed over an AMC array, are fabricated on flexible Rogers ULTRALAM 3850 and RO3003 substrates, respectively. The proposed antenna exhibits a dual band resonance and acceptable gain at a distance of 15 mm from the human body model. In-order to reduce such separation and to enhance the gain and reduce the SAR, an AMC array is utilized. Cutting the edges of the conventional square patch to a triangular-shaped patch, cutting slots in the CPW ground plane, and cutting slots and slits in the radiator, help in achieving the single band, dual-band, and lowering the second frequency, respectively. To check the SAR, the antenna is placed at different distances over a human body model with skin, fat, and muscle tissues of thicknesses of 2, 8, and 23 mm, respectively. The SAR is measured as 0.0.683 W/kg at 3.5 GHz and 0.333 W/kg at 5.8 GHz for 1 g of tissue and 0.0226 W/kg at 3.5 GHz and 0.0814 W/kg at 5.8 GHz.

#### 4.1.4. DRA-Based Flexible Antenna

A dielectric resonator antenna (DRA) is another suitable candidate for 5G applications. A dielectric resonator can replace the conventional antenna since DRAs have a high radiation efficiency and a wide bandwidth and do not experience conduction losses. [58].

**Figure 12:**
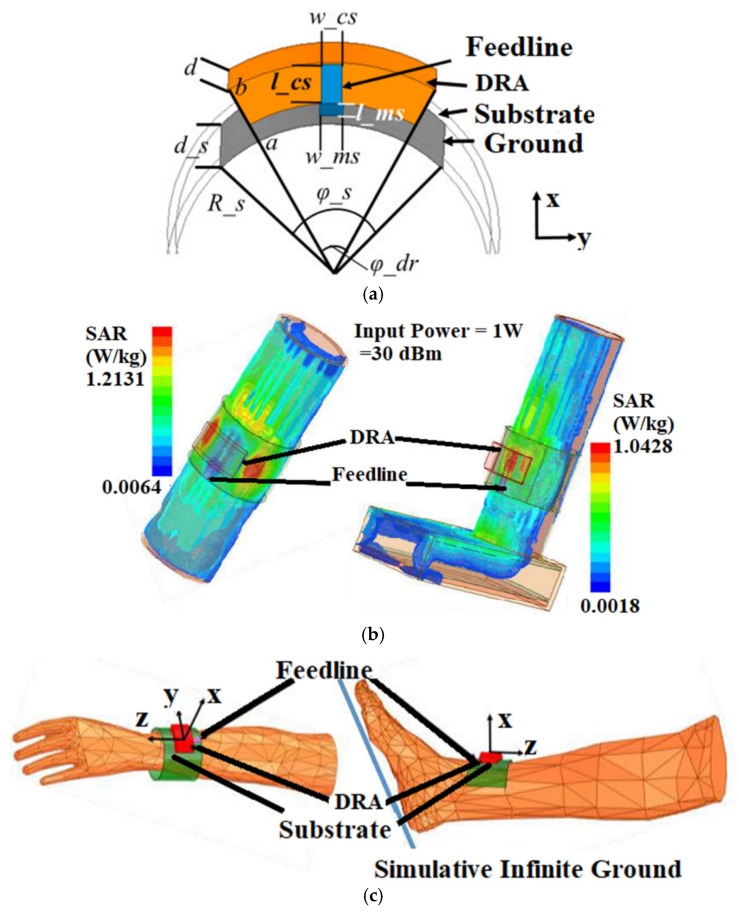
DRA antenna for sub-6 GHz applications: (**a**) Schematic; (**b**) SAR analysis on the biological tissue models; (**c**) Analysis of the DRA on a limp model. Reprinted from ref. [59].

In [59], a wearable dielectric resonator antenna in the form of a wristwatch is suggested for the 2.45 GHz ISM band and sub-6 GHz bands. As the feeding structure for the isolation, a vertical strip and a coplanar microstrip line are utilized. Figure 12a shows the schematic of the proposed antenna. To identify the conformal nature of the antenna, they are placed on the forearm and ankle model. There is a typical variation in the radiation due to the substrate radian changes. A slot is etched to suppress the substrate effect and because of the concentration of the electromagnetic energy around the slot, less backward radiation was emitted. The SAR is calculated by placing the DRA on multilayer biological tissue models with different electromagnetic properties and mass densities in each layer, as shown in Figure 12b. The average SAR of 1 g of tissue is below 1.22 W/kg. To check the performance variation when the DRA for users, a limp phantom model is considered, as shown in Figure 12c. The antenna has a simulated gain of 5.15 dBi and a measured gain of 4.6 dBi.

In another design in [60], a compact button-shaped flexible antenna using a dielectric resonator is proposed for wearable applications. The antenna is in the shape of a button which consists of a ring-shaped resonator and is attached to a ground plane. A rectangular slit cut into the ground plane can be used to couple a microstrip line for the excitation. The antenna achieved a simulated efficiency of 50% to 60% in the specified bandwidth. At an operating frequency of 5.8 GHz, the antenna gives a gain of 5.4 dB. To understand the effect of the electromagnetic waves on the human body, the antenna is placed on a parallelepiped phantom. It is observed that for 1 g of tissue, the SAR is 0.105 and for 10 g of tissue, the SAR is 0.27, which is below the specified international limits.

#### 4.1.5. Transparent Flexible Antenna

In [29], another slot-based wideband and transparent flexible co-planar waveguide antenna is fabricated on a PET substrate with a bandwidth ranging from 3.89 GHz to 5.9 GHz for the WLAN and sub-6 GHz. The antenna consists of two circular rings that are embedded in a star-shaped structure. The CPW feed contributes to a good impedance matching and smooth movement of electromagnetic waves. The change in the inner radius of the circular ring changes the reflection coefficient at lower and higher resonances with a negligible variation in the impedance bandwidth of the antenna. The antenna gave a notable gain of 3 dBi and an efficiency of 80% for the entire frequency band. The bending analysis of the antenna is performed with Styrofoam ($\varepsilon_{r}$ = 1.03) having a radius of 55 mm along the x- and y-axis. The measured reflection coefficient shows a deflection from the simulated results. However, the antenna resonates at a specific band suitable for sub-6 GHz 5G applications.

In [61], a transparent antenna that is dually polarized and fabricated on a polycarbonate material is proposed for sub-6 GHz applications. The antenna is a stacked structure that consists of a square parasitic patch on the impedance matching, whereas the cutting the corners of the driven patch and the etching of slots in the ground contribute to the port isolation and stable gain. The antenna shows a measured gain of 4.14 dBi for a frequency range of 2.8–3.8 GHz. The antenna provides an impedance bandwidth ranging from 2.76 to 4.13 GHz and a good isolation greater than 22 dB. The antenna has a directional radiation pattern at 2.9 and 3.65 GHz. The front-to-back ratio is found to be better than 15 dB in the operating band.

In another design in [62], a tri-band transparent and flexible antenna for 5G applications is presented. The proposed antenna consists of a transmission line which consists of a tree-shaped structure, with two rectangular patches. Two periodic slots are cut from the patch to achieve an additional resonance at 5.7 GHz. The reflection coefficient of the antenna shows better results when integrating the ITO than the copper substrate. The antenna covers a bandwidth ranging from 2.35–2.55 GHz, 3.75–3.9 GHz, and 5.35–5.95 GHz. The peak gains of 3.27 dBi, 9.47 dBi, and 4.47 dBi for 2.45 GHz, 3.8 GHz, and 5.7 GHz are achieved. The bending performance of the antenna is performed for different bending degrees, and it is observed that there is a shift in resonance for bending at 30° due to the change in the surface current distributions.

The summary of the single-element flexible antenna for the sub-6 GHz 5G band is given in Table 7.

### 4.2. Flexible Array Antennas for a Sub-6 GHz 5G Band

An array antenna is the combination of multiple antenna elements arranged in a particular orientation where the radiation characteristics of the antenna is determined by the orientation and the separation of the antenna elements. In array antennas, all of the antenna elements can be fed to single port. This section gives a review of various flexible array antennas for a sub-6 GHz band.

#### 4.2.1. Slot/Slit Based Flexible Antenna

**Figure 13:**
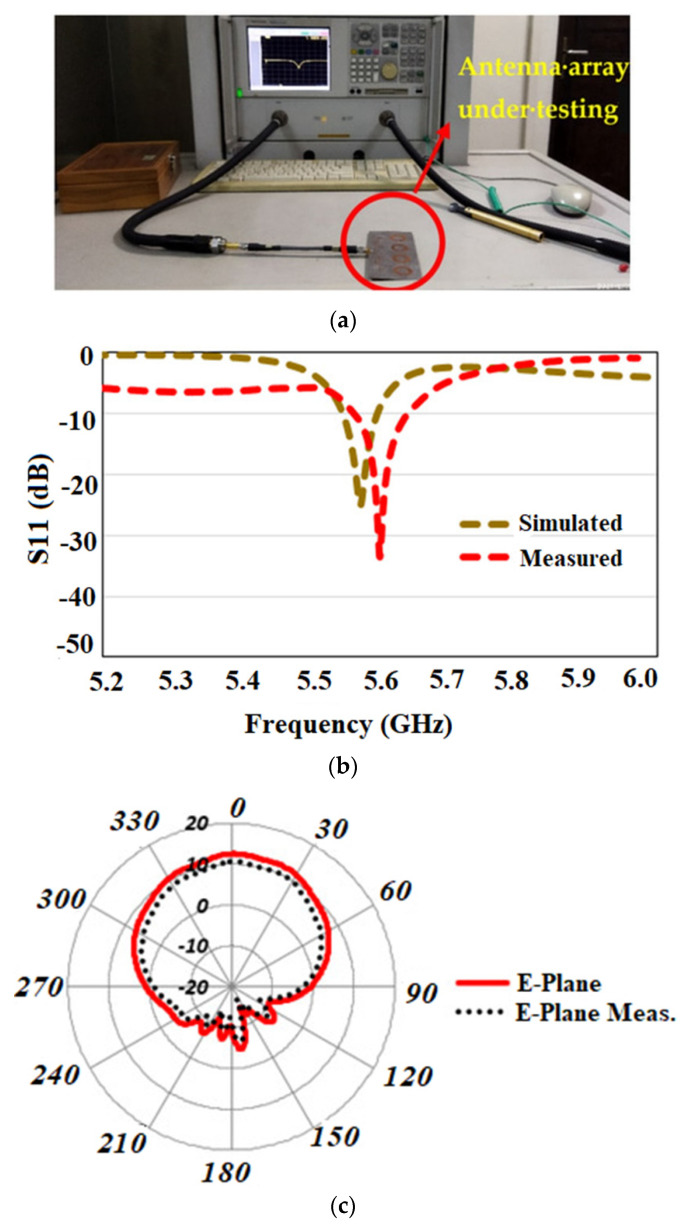

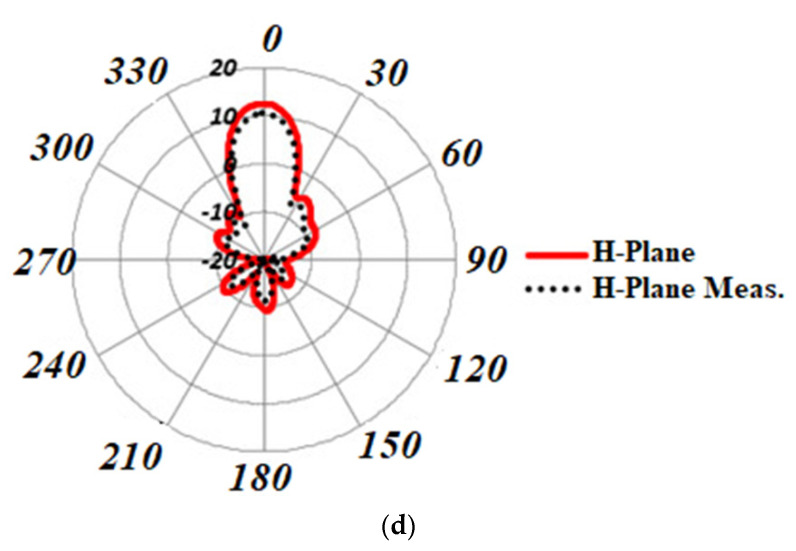
Slot based array antenna for sub-6 GHz 5G: (**a**) Antenna under testing; (**b**) S-parameter; (**c**) Radiation pattern along the E-plane and (**d**) H-plane. Reprinted from ref. [63].

In [63], a flexible array antenna is proposed for sub-6 GHz 5G applications. The antenna consists of a slot-integrated circular patch that is embedded on a semi-flexible RT5880 substrate. Figure 13a shows the proposed antenna under testing. The antenna covers a bandwidth ranging from 5.55–5.6 GHz, as shown in Figure 13b. A corporate fed array where each element is placed symmetrically to each other is proposed.

The array has a gain of 12 dB and achieved the isolation of more than −30 dB for a two-port configuration. The array achieves an efficiency of 80% within the band of operation. The SAR analysis of the proposed array antenna is conducted with a body model that consists of different layers exhibiting a different conductivity as well as relative permittivity values and is 0.0989 W/kg for 10 g of tissue which is well below the international standards. The array antenna has a directive radiation pattern, as shown in Figure 13c,d.

Another slot-based array is proposed in [64], where the antenna array consists of a slot incorporated in the radiating patch and a parasitic patch towards the center that provides proper isolation. The array consists of graphene and semi-flexible epoxy fiber as the radiating patch and substrate, respectively. A partial ground plane is made to achieve the bandwidth enhancement. The antenna operates for a bandwidth of 2.21–5.13 GHz. A measured gain value of 2.73 and 3.744 dBi for 2.40 and 4.0 GHz is achieved. The antenna has an efficiency of 78%. The array antenna shows a directional radiation pattern at 2.45 and 4.0 GHz. The bending analysis of the antenna is performed along different angles and it is seen that there is a slight fluctuation in the reflection coefficient at a bending angle of 14° in the E-plane and 18° in the H-plane, respectively.

#### 4.2.2. Metamaterial-Based Flexible Antenna

A flexible antenna array that is wideband and has a unidirectional radiation pattern is utilized in many medical applications in which the signal penetration can be focused on the targeted diagnosis region. One such wideband flexible array is proposed in [65] for wearable head imaging systems. The antenna comprises a meander line, a thick copper ground plane with 50 Ω coaxial feed. The grounding of the cascaded meander line with the help of via introduces an additional inductance with which an enhanced bandwidth to 155% covering 0.45–3.6 GHz is achieved. A 16-element array is constructed to test to validate the results of the EM head imaging system. To investigate the bending performance, the antenna was bent using a multi-tissue model along different radii of r = 70, 60, 50, 40, 30, and 20 mm. It is observed that at the r = 70 mm, the array shows a similar antenna performance as that of a planar case. The radiation pattern over lower frequencies of 0.5 and 1 GHz shows a more unidirectional kind of pattern with a half power beam width of approximately 90°. The SAR value for the operational bandwidth of 0.45 to 3.6 GHz is 0.2 W/kg for 10 g tissue which is under IEEE defined limit.

#### 4.2.3. Transparent Flexible Antenna

**Figure 14:**
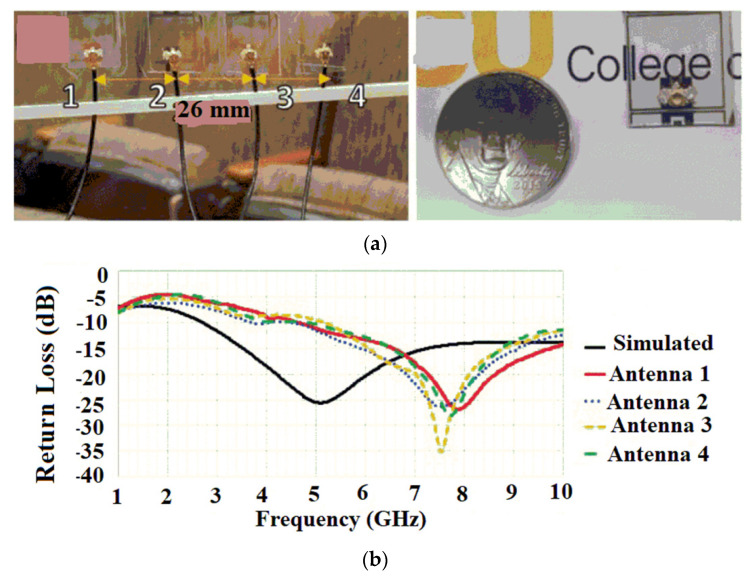
Transparent array antenna for 5G communication: (**a**) Fabricated GZO array (**left**) and single element (**right**); (**b**) Simulated and measured results of all of the fabricated antennas. Reprinted from ref. [66].

An optically transparent bowtie-slot array antenna for mid-band 5G applications is proposed in [66], where the antenna consists of a gallium-zinc oxide (GZO) conductive film embedded on a transparent Rogers 6006 substrate, as shown in Figure 14a. Four antenna elements are designed, and the elements of the array antenna are placed at 26 mm to provide better isolation between the elements. The array antenna could improve the gain to 0.34 dB from −10 dBi gain achieved with a single antenna element. Compared with the fabricated copper antenna counterpart, the optical array antenna contributes to a smaller gain value due to the low conductivity values of the transparent conductive materials. The array antenna is also fabricated with a copper film as the conductive material to compare the performance of the array with the GZO array. It is observed that the proposed antenna contributed to an enhancement of the bandwidth ranging from 4.22 to 10 GHz, as shown in Figure 14b. The array antenna provides a good optical transparency of 90%.

### 4.3. Flexible MIMO Antennas for Sub-6 GHz 5G Band

**Figure 15:**
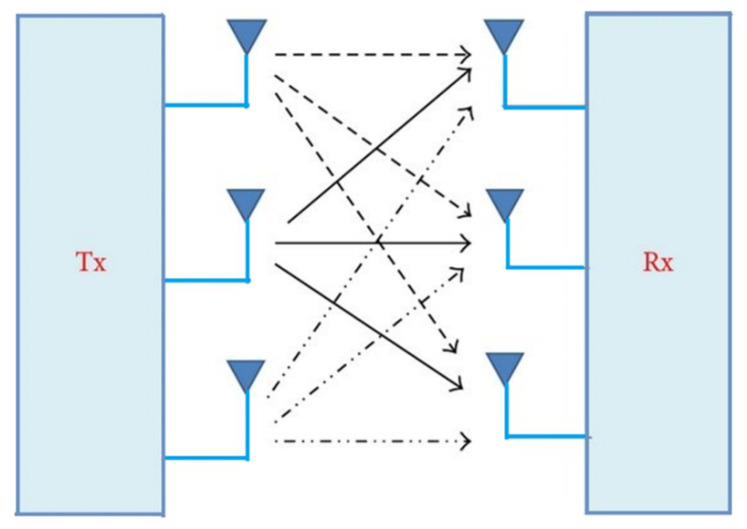
The basic structure of MIMO. Reprinted from ref. [67].

A Multiple-Input Multiple-Output system is a promising technology that can be used for the recent generation of communication systems since MIMO contributes to a higher spectral efficiency, a larger coverage area, and an increased system capacity. By utilizing numerous antennas at the transmitter and receiver, which allow different signal paths to deliver the same information, the channel’s capacity can be improved. The MIMO technology makes use of multi-path environments providing the receiver with multiple versions of the same signal. Over the past few years, MIMO techniques for wireless communication have been researched to achieve the considerable capacity improvements required to enable a high-rate communication. Figure 15 shows the basic structure of the MIMO system [67]. The single patch element antennas discussed in the previous section are still not sufficient to meet the channel capacity. Therefore, the MIMO technology is a suitable candidate to be utilized for the enhancement of channel capacity in the sub-6 GHz 5G band.

In MIMO antennas, multiple antenna elements are oriented with an acceptable isolation between the antenna elements and each input antenna is fed to a separate port and also, the ground planes of the individual elements are connected together. When multiple antennas are placed at a distance less than the quarter wavelength, then there is an interference between the elements of the antenna, that affects the efficiency of the MIMO system. This is termed mutual coupling. Closely oriented antenna elements will lead to a high coupling between the antenna elements that have to be reduced by the proper orientation of the antenna after a very proper analysis on how exactly the antenna elements could be placed. To provide better isolation between the individual elements in a MIMO system and to provide better isolation, there are various decoupling structures used, such as parasitic elements [68], a defected ground plane structure (DGS) [69], a neutralization line (NL) [70], metamaterial loading (MTM) [71] and so on.

**Figure 16:**
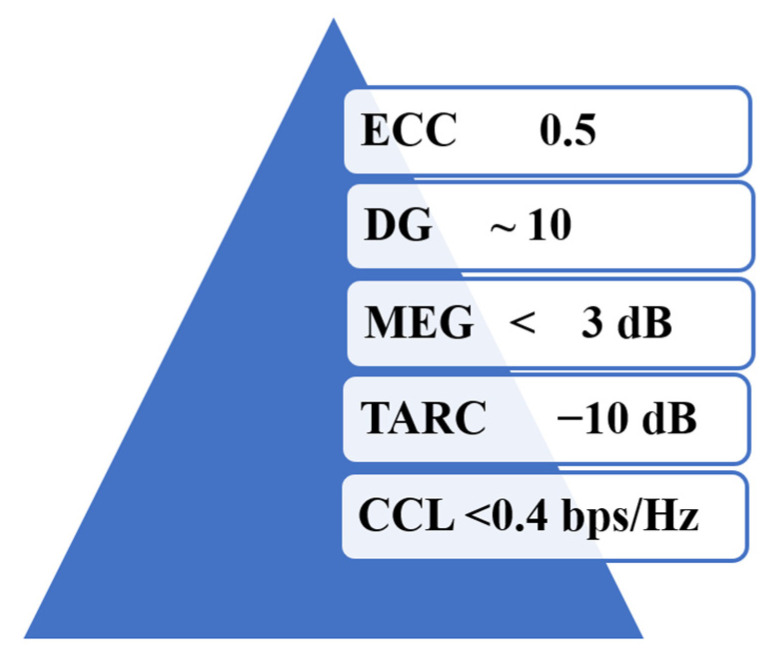
Threshold values of the different MIMO diversity parameters. Reprinted with permission from ref. [72]. Copyright 2021 Elsevier.

To ensure the performance of the MIMO antenna, several performance metrics have to be analyzed. The key performance metrics in the analysis of the MIMO performance are the envelope correlation coefficient (ECC), the diversity gain (DG), the mean effective gain (MEG), the total active reflection coefficient (TARC), and the channel capacity losses (CCL). All of these parameters must be within the threshold level to ensure a good MIMO performance [72]. The threshold values of the abovesaid diversity parameters are as shown in Figure 16.

#### 4.3.1. Slot/Slit-Based Flexible Antenna

**Figure 17:**
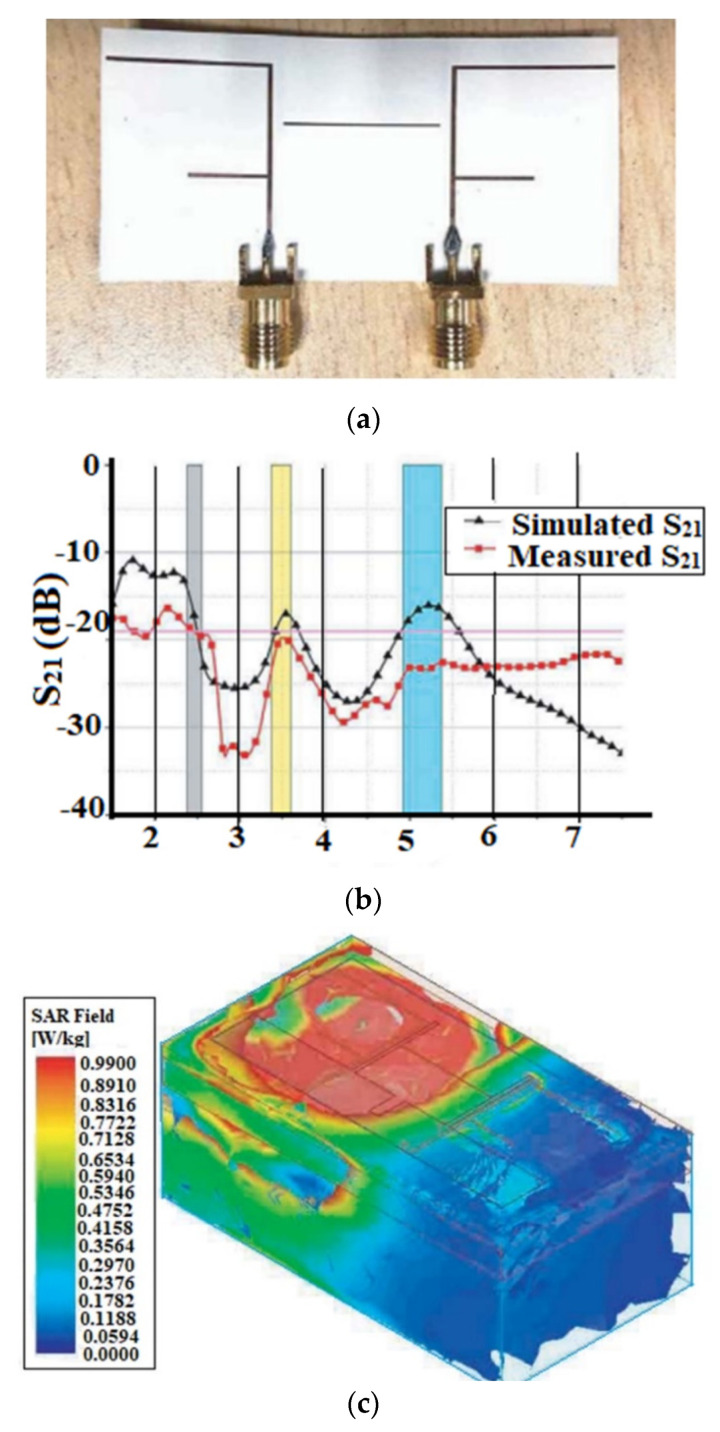
Triband MIMO antenna: (**a**) Fabricated prototype; (**b**) S-parameter; (**c**) SAR distribution in 10 g of tissue at 3.5 GHz. Reprinted from ref. [73].

A tri-band MIMO antenna is fabricated on a flexible LCP substrate [73]. The MIMO structure consists of an inverted-F branch that provided the two lower frequency bands, as shown in Figure 17a. To achieve the third band, and to have a better isolation between the elements, an I shaped structure and a slot are introduced in the ground plane that gives better isolation of −10 dB. The measured impedance bandwidth is 2.38–2.55 GHz, 3.37–3.60 GHz, and 4.92–5.37 GHz and the isolation achieved is greater than 19 dB for all of the working bands, as shown in Figure 17b. The bending analysis of the MIMO antenna is performed, and it is found that for both the E-plane and the H-plane, there is negligible impact on the isolation of the antenna. The SAR analysis is performed by keeping the antenna at various distances from the human tissue model consisting of different layers of skin, fat, and muscle. The minimum SAR values of 0.64 and 0.99 for 2.45 GHz and 3.5 GHz for 10 g of tissue are achieved at 1mm from the human body. Figure 17c shows the SAR distribution in 10 g of tissue at 3.5 GHz.

**Figure 18:**
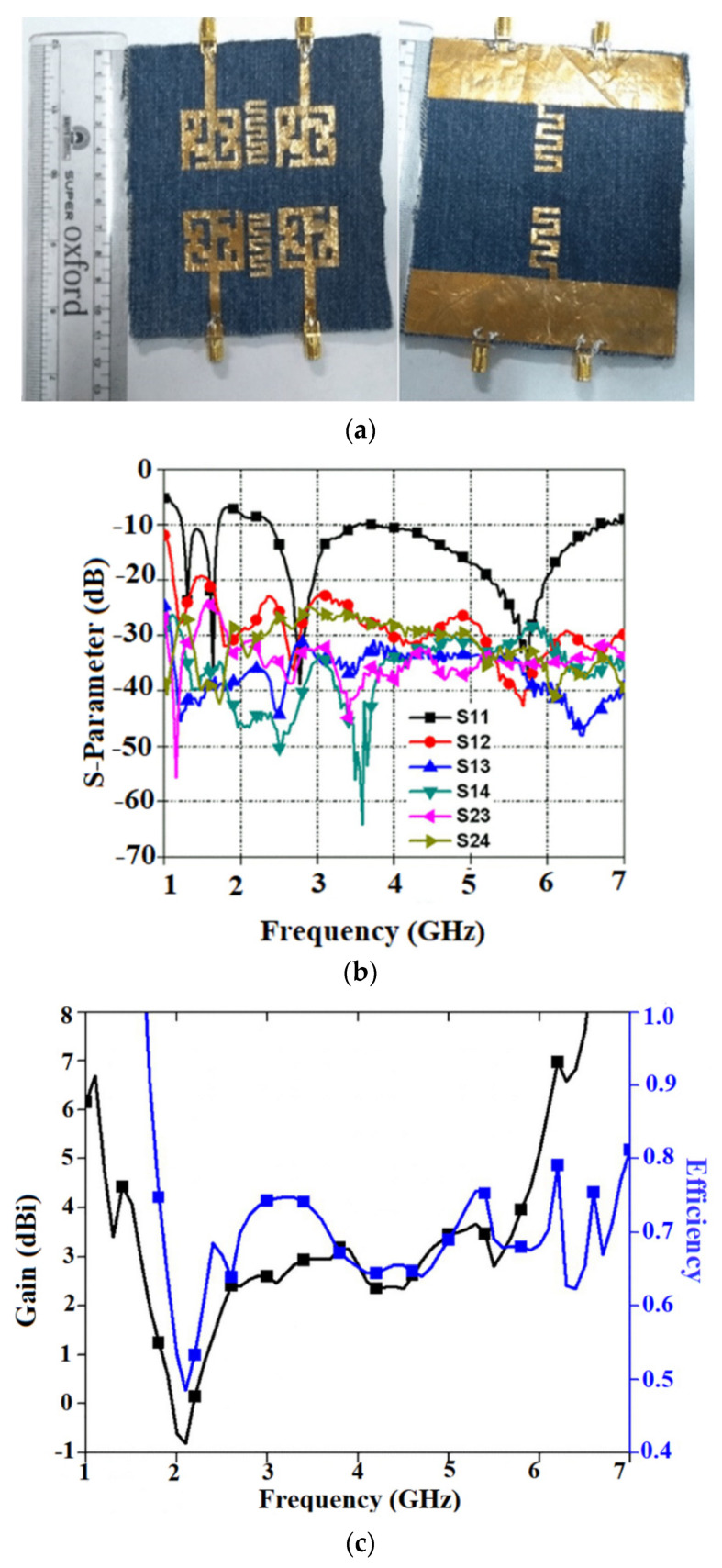
Dual-polarized slotted textile MIMO antenna: (**a**) Antenna prototype; (**b**) S-parameter of the antenna; (**c**) Gain and efficiency. Reprinted with permission from ref. [74]. Copyright 2020 John Wiley & Sons, Inc.

Fabric substrates are widely used in the design of several MIMO systems due to their low dielectric constant values. In [74], a wideband dual-polarized textile antenna is designed on a jeans substrate, as shown in Figure 18a. The antenna has a bandwidth ranging from 1.5 to 3.8 GHz and 4.1 to 6.1 GHz. Figure 18b shows the measured S-parameter of the antenna. The antenna provides an efficiency of 70% with a peak gain of 4 dBi at the desired frequency range, as shown in Figure 18c.

The mutual coupling is decreased by implementing a meander line in the ground plane. Introducing another meander line on the top layer enhanced the diversity performance of the antenna in the specific frequency ranges. The antenna exhibits a dipole-type pattern in the E-plane and an omnidirectional type of pattern in the H-plane. The SAR value is below 1.6 W/kg which is below the international standards.

Another MIMO antenna design on a jeans substrate with a different isolation technique is proposed in [75], in which an E-stub is inserted between the radiators to improve isolation, and the antenna is made up of two square radiators with slots running vertically and horizontally on the front end. A partial ground plane has been etched to increase the antenna’s bandwidth. The introduction of stubs contributes to a minimal mutual coupling between the radiators. A SAR analysis of the antenna is carried out to check the suitability of the antenna in wearable applications. The analysis is carried out using a human body model consisting of different layers of tissues. The antenna is placed at different distances from the model. The minimum average SAR over the 1 g volume of tissue at 3.5 GHz is found to be 0.62 for the human body model, placing the antenna at a distance of 5 mm from the model. The antenna diversity parameters are estimated and showed good results suitable for the MIMO.

#### 4.3.2. DGS-Based Flexible Antenna

**Figure 19:**
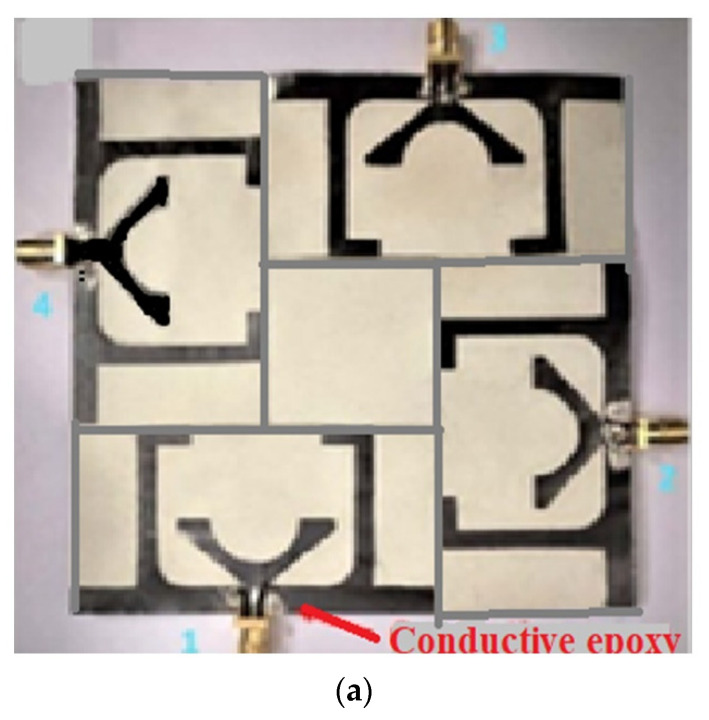

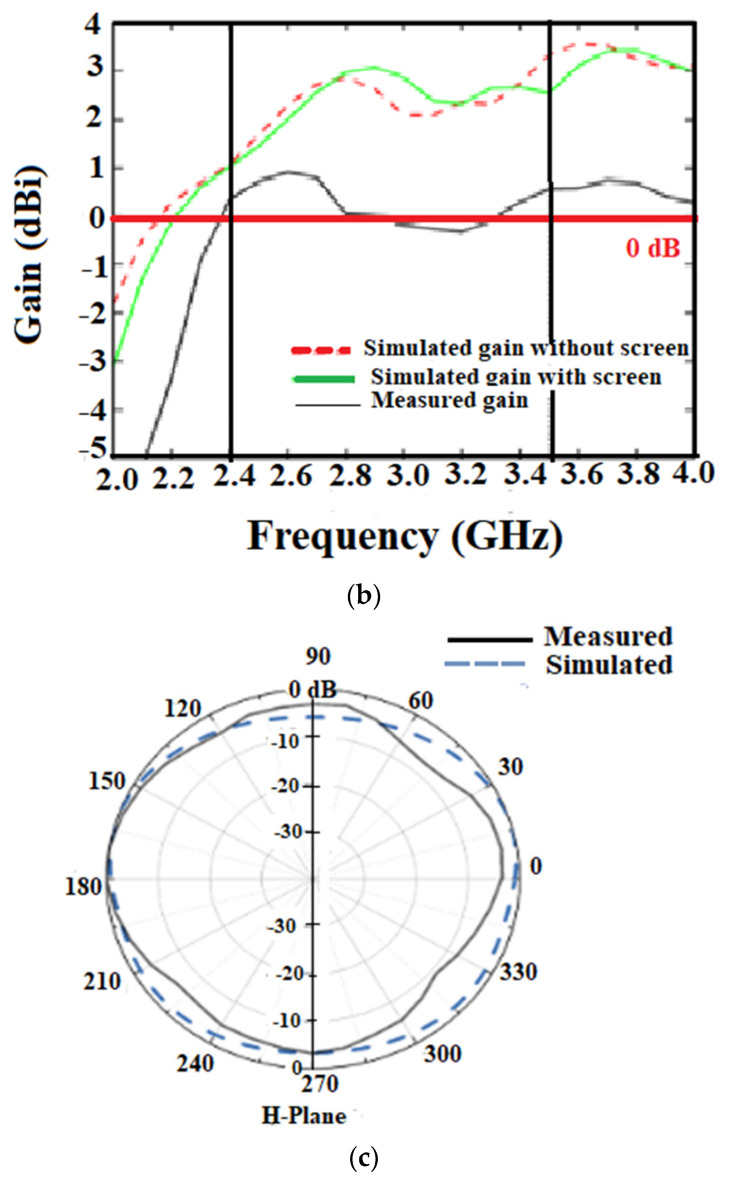
Wideband MIMO on a paper substrate: (**a**) Antenna prototype; (**b**) Gain analysis; (**c**) Radiation pattern at 3.5 GHz. Reprinted from ref. [76].

Defected ground plane structures are found to enhance the gain and provide better isolation among the elements. In [76] a wideband MIMO is fabricated on a flexible paper substrate, as shown in Figure 19a. The inverted L-shaped stubs that connect the ground plane enhance the gain and decrease the coupling effect of the antenna elements. The antenna has a CPW feed with a bandwidth that of 2.22 to 3.85 GHz. A measured gain of 0.4, 0.56, and 0.92 dBi at 2.4, 3.5, and 2.6 GHz, respectively, is achieved suitable for 5G mid-band applications. as shown in Figure 19b. There is a negligible effect on the resonant frequency with the bending analysis, but the S-parameter is more deviated in the horizontal bending than in the vertical bending due to the deformation of the ground extension stub. The antenna shows a directional radiation pattern, as shown in Figure 19c.

**Figure 20:**
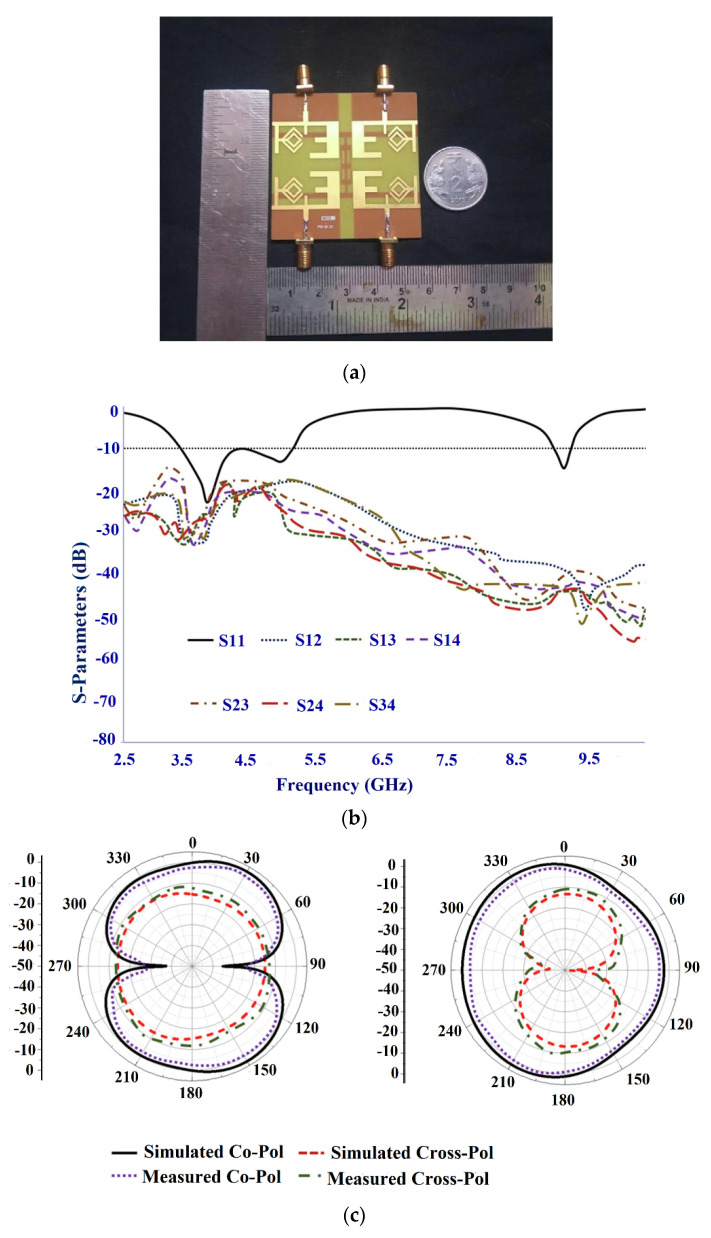
Four element antenna with a truncated ground: (**a**) Fabricated prototype; (**b**) S-parameter; (**c**) Radiation pattern. Reprinted with permission from ref. [77]. Copyright 2022 Elsevier.

In another design in [77], a flexible four-port and four-element flexible MIMO antenna with the truncated ground are proposed for sub-6 GHz and X-band applications. The antenna consists of an inverted E and inverted L-shaped conductive material on either side of the central feed line, as shown in Figure 20a. The bottom part consists of a ground plane which is truncated, and a vertical stub placed on one side for achieving the wideband performance for the sub-6 GHz band. The flexible four-port resonator has a connected ground with an I-shaped strip with a slotted rectangular stub for better isolation of more than 20 dB. The antenna achieves a 70% efficiency and 4 dBi gain covering 3.34–5.0 GHz and 8.9–9.2 GHz for the sub-6 GHz and X-band applications. Figure 20b shows the S-parameter of the proposed antenna. By bending the antenna around a cylinder with a 20 cm radius along the x- and y-axes, the antenna’s bending analysis is carried out. The antenna demonstrates a favorable gain and ECC under the bending scenarios, as well as good transmission and scattering curves, making it a suitable choice for the aforementioned applications. The antenna gives a directional and omnidirectional pattern in the E-plane and H-plane, respectively, as shown in Figure 20c.

#### 4.3.3. Metamaterial-Based Flexible Antenna

In [78], a compact and flexible MIMO antenna is proposed. The structure consists of a T-shaped monopole with a negative permeability (MNG) unit cell to decrease the mutual coupling. The proposed unit cell called the bridge rectangular split-ring resonator (BS-SRR) provides a negative permeability over a certain bandwidth of the antenna. The antenna is fed by a microstrip feed line with a partial ground plane to enhance the bandwidth of the antenna. The antenna operates at 4.9 GHz with a bandwidth of 695 MHz. To check the flexibility of the antenna, the antenna is bent over a cylindrical foam of a radius of 13 mm, and it is observed that there is no significant change in the reflection coefficient of the antenna. The antenna gives a gain of 2.2 dB with an efficiency of 70.7%. The antenna gives an omnidirectional radiation pattern in both planes.

**Figure 21:**
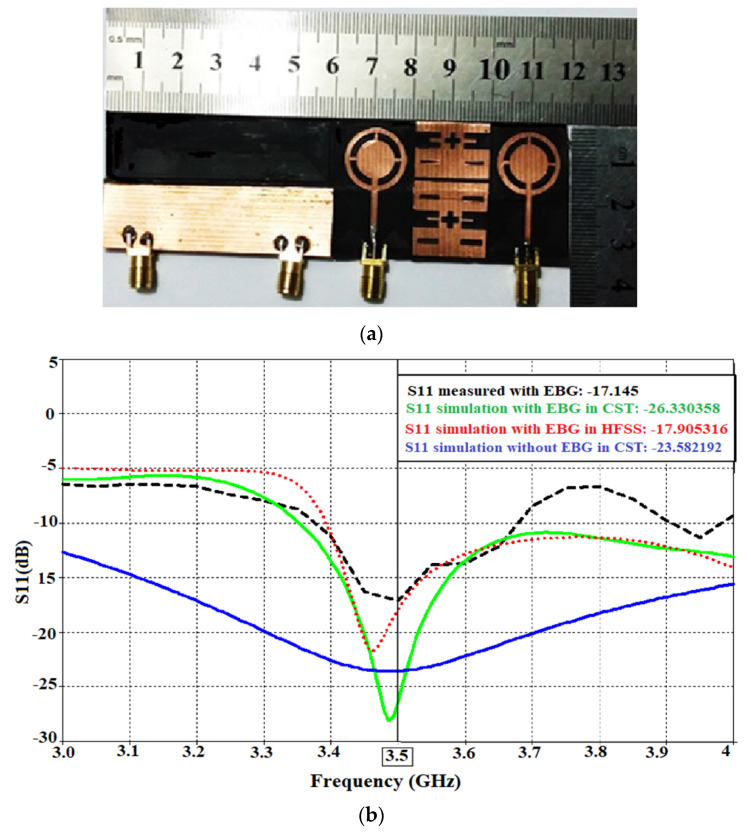
EBG-based MIMO antenna: (**a**) Antenna prototype; (**b**) S-parameter. Reprinted from ref. [79].

In [79], a flexible MIMO antenna with a circular slotted patch is proposed to work at 3.5 GHz, as shown in Figure 21a. A partial ground plane is implemented to enhance the bandwidth of the antenna. The mutual coupling of the antenna is reduced by −11.6 dB, by the utilization of an electromagnetic band gap (EBG). The structure used as metamaterial reduces the mutual coupling by creating a band gap at the required frequency to suppress the surface currents that cause the mutual coupling. Moreover, with the introduction of the EBG structure, the gain and the efficiency of the antenna are improved by 1.074 dB and 2.4%, respectively. The bending analysis of the antenna is performed using a cylindrical foam with a radius of 13 mm. There is no noticeable change in the values of S_11_ and S_21_ for the desired frequency of operation. Figure 21b shows the S-parameter of the antenna.

#### 4.3.4. DRA-Based Flexible Antenna

**Figure 22:**
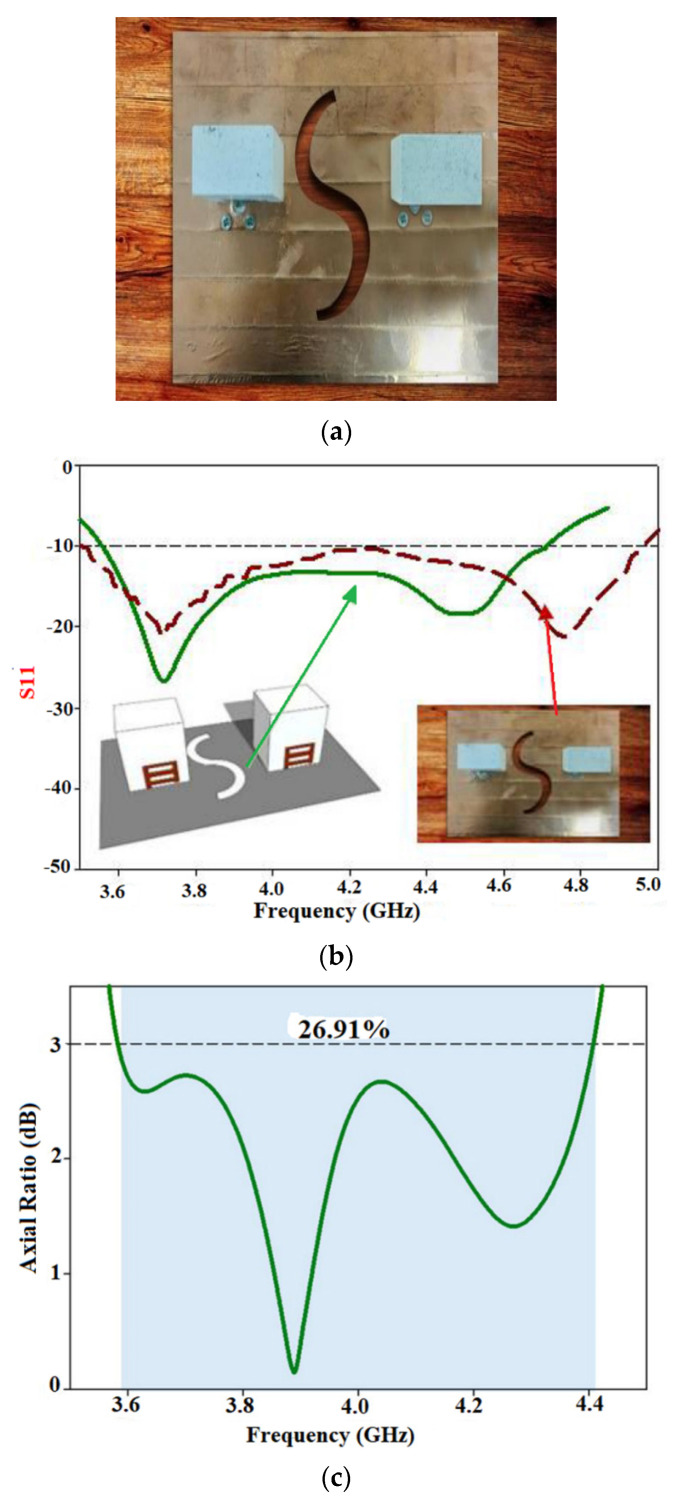
DRA MIMO antenna for the sub-6 GHz 5G application: (**a**) Antenna prototype; *(***b**) Simulated and measured S-parameter; (**c**) Axial ratio of the proposed antenna. Reprinted from ref. [80].

In [80], a circularly polarized rectangular dielectric resonator antenna with a ceramic material is proposed for mid-band 5G applications, as shown in Figure 22a. The antenna is compact but lacks the desired flexibility, due to the low flexibility of the ceramic material. However, a conformal metal strip is used to achieve the circular polarization. The antenna has a bandwidth of 3.57–4.48 GHz which makes it suitable for a specific application, as shown in Figure 22b. The axial bandwidth of almost 28.33% is achieved, as shown in Figure 22c. A better isolation of −28 dB between the elements is achieved using an “S” shaped defective ground plane structure. An average gain of 6 dBic is achieved.

#### 4.3.5. Transparent Flexible Antenna

A transparent MIMO antenna has come to the frontline these days because of its good visual clutter. A transparent two-element circular patch antenna is proposed in [81], utilizing transparent AgHT-8 and plexiglass as the conducting and substrate materials, respectively, which utilizes a radiating structure in the form of a solid cylinder enclosed in a hollow cylinder that connects a stub. The required frequency is achieved by modifying the ground plane to a partial ground.

**Figure 23:**
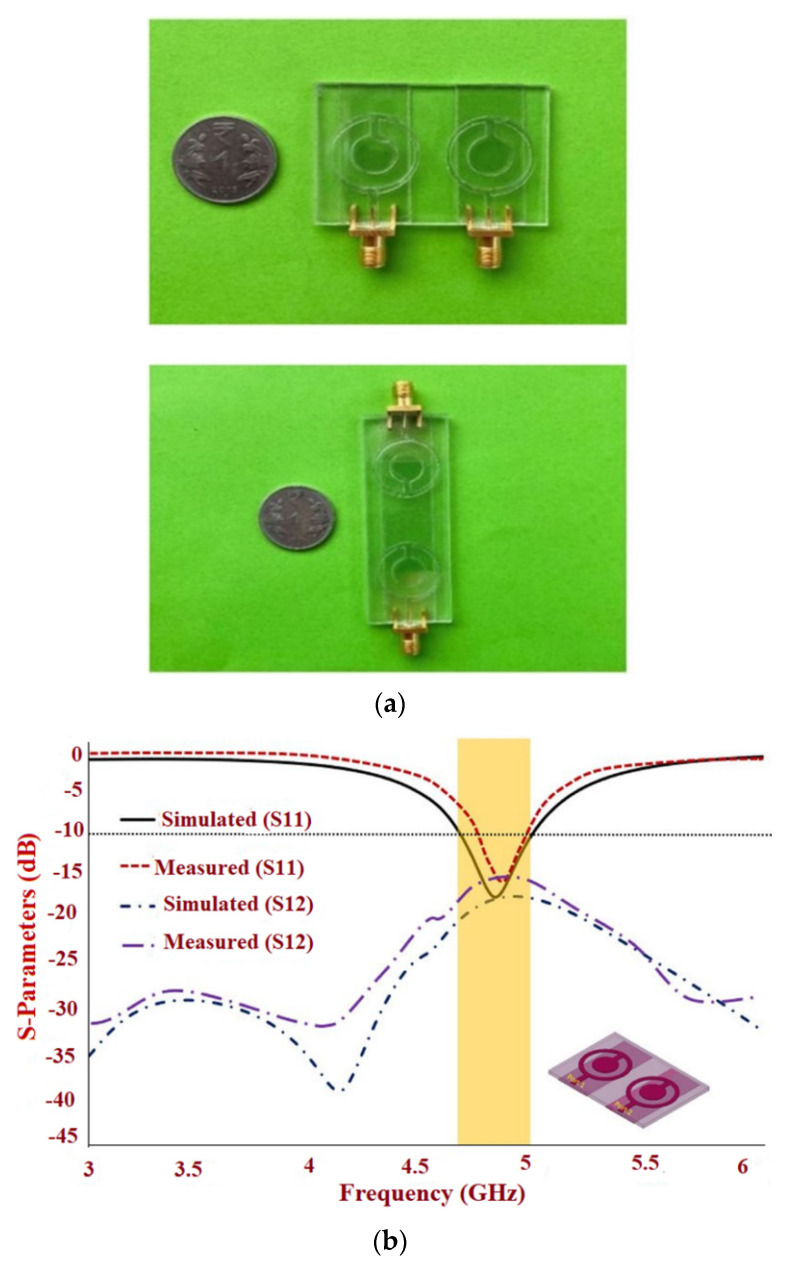

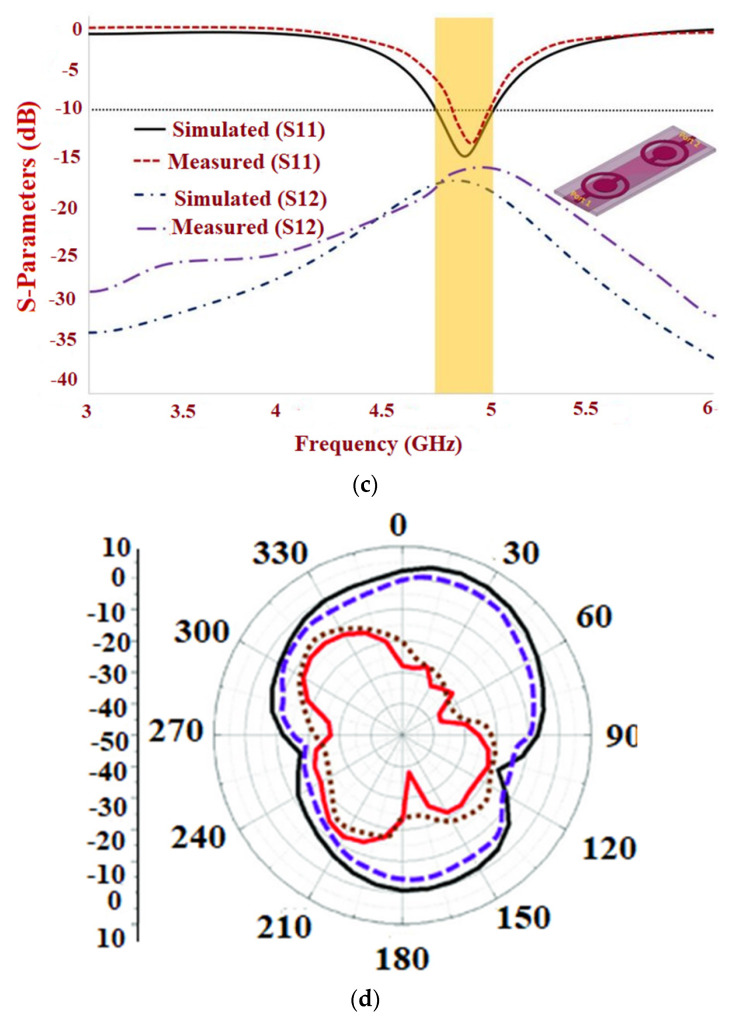
Transparent MIMO antenna: (**a**) Fabricated prototype horizontal (Case 1) and a vertical (Case 2) orientations; (**b**) S-parameter of Case 1; (**c**) S-parameter of Case 2; (**d**) Radiation pattern at 4.81 GHz (Case 1). Reprinted from ref. [81].

Two MIMO configurations are proposed. Case 1 consists of a separate ground configuration having a similar orientation of the elements, whereas Case 2 consists of oppositely arranged antenna elements with a connected ground, as shown in Figure 23a. To achieve the improved impedance matching and isolation, the partial ground is made for Case 1 and a connected ground with 15 mm from the individual element is carried out for Case 2, which give an isolation of greater than 17.10 dB for Case 1 and 17.38 dB for Case 2, respectively. The impedance bandwidth ranging from 4.65 to 4.97 GHz for Case 1 and 4.67 to 4.94 GHz for Case 2 is achieved, as shown in Figure 23b,c. The peak gain of 1.83 dBi and 1.65 dBi is achieved with an efficiency of 53% and 59% for Case 1 and Case 2, respectively. A directional radiation pattern is achieved, as shown in Figure 23d.

**Figure 24:**
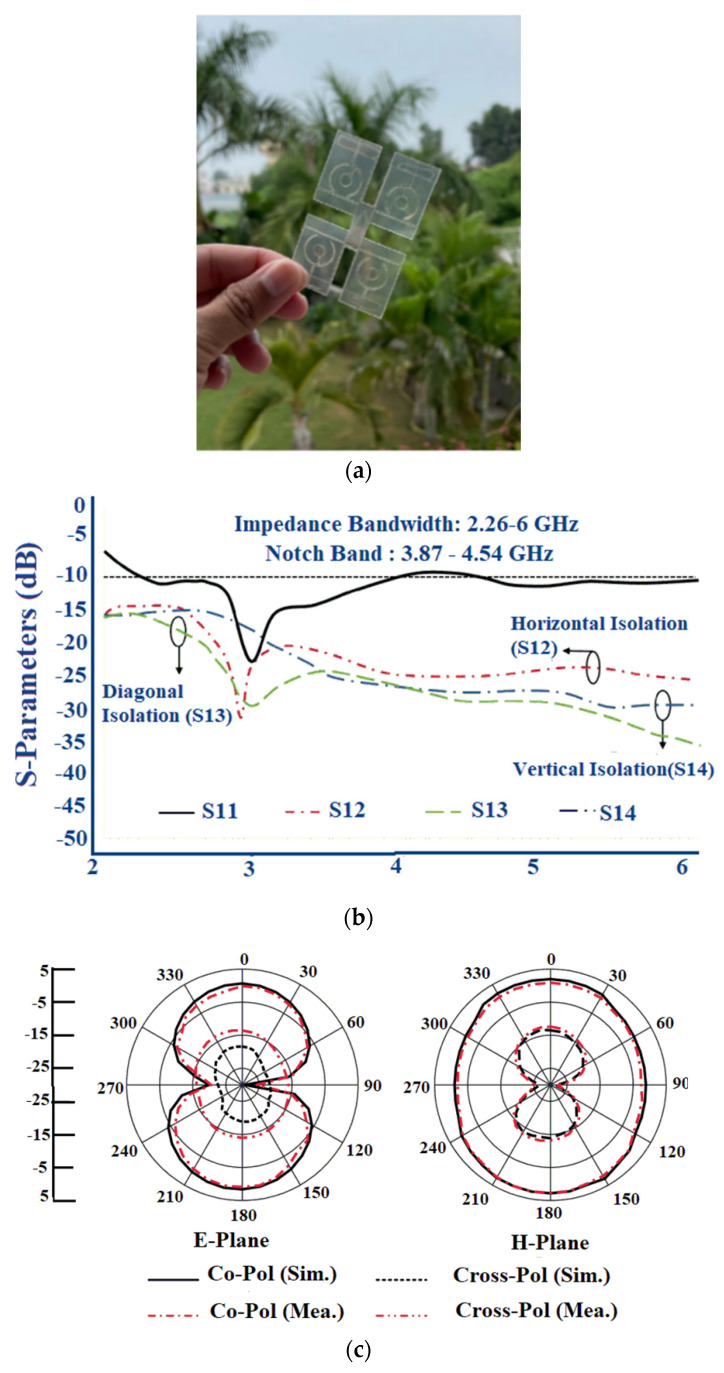

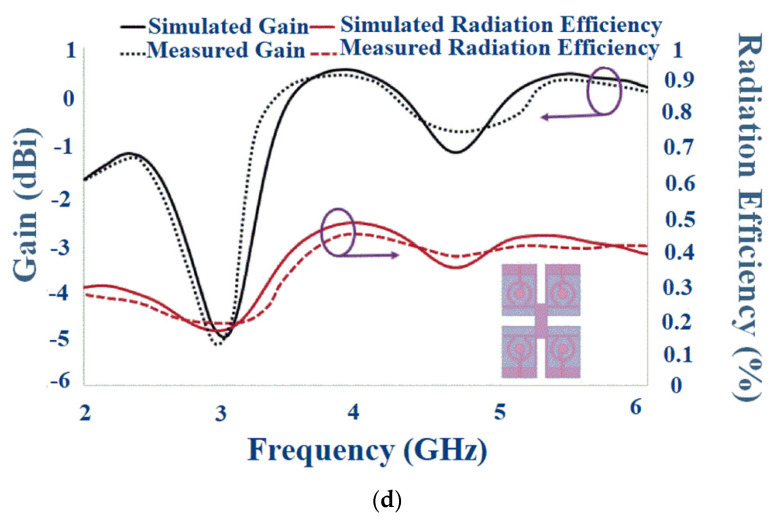
Transparent antenna with the connected ground: (**a**) Fabricated prototype; (**b**) S-parameter; (**c**) Radiation pattern; (**d**) Gain and efficiency of the proposed antenna. Reprinted from ref. [82].

In another design in [82], a wideband flexible transparent four-element MIMO is utilized for the sub-6 GHz 5G applications. The radiating part consists of a circular stub-loaded C-shaped resonator with an L-shaped partial ground embedded on either side of the substrate. The appropriate bandwidth and the reflection coefficient are produced by the L-shaped resonator integrated with a partial ground plane. To model the MIMO antenna with a connected ground plane, the conductive oxide is added to the bottom portion of the substrate which connects all of the ground planes of the four elements. Figure 24a shows the fabricated antenna prototype. The antenna provides a wide bandwidth of 2.21–6 GHz with an isolation greater than 15 dB between the elements, as shown in Figure 24b. The radiation pattern of the fabricated antenna gives a directional radiation pattern as in Figure 24c.

The maximum gain of 0.5 dBi with an efficiency of 41% is achieved. The bending analysis of the MIMO antenna is carried out using a Styrofoam cylinder of the radius with a prescribed bending angle. It is observed that the maximum gain and efficiency when the antenna is bent along the x-axis, was 0.47 dBi and 34%, respectively, and 1.07 dBi and 33%, respectively, for y-axis bending, as shown in Figure 24d. The summary of the flexible MIMO antenna for sub-6 GHz 5G band is given in Table 8.

## 5. Challenges in Designing a Flexible Antenna

This session depicts the various challenges in designing the flexible antenna for various applications with respect to the substrate selection, effect of the human body on the antenna performance, deterioration in the gain, and the miniaturization of the antenna.

### 5.1. Substrate Selection

The design of a flexible antenna for different applications has a patch that is embedded on a flexible substrate. The choice of proper conductive and substrate materials is the main consideration in the design of any flexible antenna. Substrates with low dielectric constants, such as fabrics, would reduce the dielectric loss and the surface waves are negligible, thereby improving the bandwidth. However, fabrics are filled with air gaps and are highly prone to absorb moisture which degrades the antenna performance. Moreover, the substrate having a relatively low dielectric constant increases the patch size. For size reduction of such an antenna, techniques, such as shorting pins, slot-loading, etc., can be implemented. The choice of proper substrate materials that exhibit good electrical and mechanical properties and which offers a good antenna performance is thus a primary parameter that has been considered while designing any flexible antenna.

### 5.2. Effect of the Human Body on the Antenna Performance

The performance of the antenna deteriorates as it is brought into proximity to the body. The presence of lossy human tissues with varying dielectric constant values, causes the antenna’s effective length to vary, which causes detuning [45]. A SAR analysis with the proper estimated distance of the antenna from the human body is another main consideration that has to be taken care of when the antenna is designed for WBAN applications. A SAR analysis has been conducted for 1 g and 10 g of tissue [83] by keeping the antenna at various distances away from the human body. It was observed that as the distance of the placement of the antenna from the human body increases, the corresponding SAR value decreases. Thus, the optimal distance measurement of the antenna should be considered so that the SAR does not cross the permissible international standards. The SAR values corresponding to different distances from the human body for 1 g and 10 g of tissue is shown in Table 9 [83].

The flexible antenna shows a difference in its behavior on the placement to different parts of the human body, due to the variation in the dielectric values of the tissues. Therefore, the antenna characteristics should be analyzed in various positions of the human body. The change in the radiation efficiency of a wearable antenna kept in various parts of the body and the maximum possible power for a designed flexible antenna at different locations of the body at 2.4 GHz is given in [10]. A radiation efficiency of an average 50% was achieved while positioning the antenna at different body locations, such as the arms, thigh, leg and on the back. A maximum input power of 0.34 W/kg at the arm position and 0.267 W/kg at the leg position fulfilled the safety standards by the EU. Therefore, the positioning of an antenna at different parts of the body and its effect on the antenna performance are other main considerations that have to be addressed for the design of any flexible antenna.

### 5.3. Deterioration in the Gain

Some flexible antennas contribute to a low gain. The gain of such an antenna should be improved using various techniques including artificial magnetic conductors, metamaterial integrated surfaces, etc. [84]. For applications involving long-distance communication, the flexible antenna should show a comparatively high gain. So, the high gain antenna is a design consideration in the implementation of a flexible antenna. Other gain enhancement techniques are EBG, FSS, DGS, etc. The use of the defected ground plane makes variations in the antenna parameters since the antenna’s ground plane comes in close contact with the body. However, the DGS integrated with a reflector can be used to increase the gain of the wearable antenna.

### 5.4. Miniaturization of an Antenna

The increase in the size of the flexible antenna may cause discomfort in the user wearing the antenna. So, a compact flexible antenna is needed especially in medical applications, and still maintaining the expected radiation characteristics is a challenge. Miniaturization techniques including applying slots, using substrates with higher dielectric constants, using fractal shapes, etc., can be used to reduce the size of the antenna and thus make it useful for wearable applications.

## 6. Discussion

A brief discussion about the different sections explained in the article is presented here. The focus of the entire paper is to give an insight into the new research that takes place in the field of flexible antennas in 5G that have grabbed the attention in the past decade. The released sub-6 GHz 5G band is the suitable choice for further investigation in the field of 5G since the band can be utilized not only for mobile applications but also in other fields including the military, health monitoring, aircraft applications, wireless body area networks, and so on with easy deployment. As far as flexible antennas are concerned, the choice of proper substrate materials and conductive materials is very crucial as they affect the performance of the antenna. In recent years, a novel Ti3C2Tx/PVDF composite with a strong negative permittivity has become increasingly popular in applications where it can replace conductive textiles that are prone to deformation [21]. Due to their excellent mechanical and electrical characteristics, the polymer-based substrates are a promising choice for the implementation of flexible antennas. Polyethylene terephthalate is used frequently in recent years due to its transparency, and good thermal and electrical properties [29]. Flexible antennas used in 5G need antennas with a positive gain and a directional radiation pattern.

A bending analysis is one of the main studies that is needed in the flexible antenna since the bending of the antenna varies the effective length of the antenna and thus varies the resonant frequency [14]. We have reviewed several flexible antennas for the 5G sub-6 GHz mid-band in this paper and have discussed the bending analysis and it is evident that there is a variation in the resonant frequency of the antenna due to bending [46,47,48]. However, the bending of the antenna across a higher radius showed a similar result in the reflection coefficient similar to the planar structure [52]. Therefore, the antenna design has to be optimized in such a way that the effect of bending has a negligible effect on the antenna characteristics.

The specific absorption rate is a very crucial term as far as WBAN applications are considered. The distance of the antenna position from the human body has a significant effect on the antenna characteristics. The SAR value decreases as the distance of the antenna from the human body increases [82].

The article has reviewed several single elements, arrays, and MIMO flexible antennas for a sub-6 GHz 5G band. Slot-based antennas are discussed in [46,47,48]. Slot elements can enhance the bandwidth of the antenna [45], contribute to a better gain [48], and also be utilized for creating band notches [47]. In [50], the multiband was achieved by the implementation of strips with the radiating patch.

The DGS-based single elements, as well as the MIMO antennas, are reviewed in the article for sub-6 GHz applications. The defective ground structure has increased the bandwidth as well as the efficiency of the antenna [51]. The DGS can increase the bandwidth of the antenna by around 117% without affecting the antenna size [52]. A large ground plane increases the mutual coupling of the antenna and reduces the isolation. The operating bandwidth and radiation properties, when used in a MIMO, are affected by asymmetric alterations made to the ground plane, which create an asymmetric current distribution. Thus, a partial ground or defected ground plane can be used. Ground planes can shield the human body from harmful radiation and also reduce the SAR [53].

Metamaterial-based antennas have also been used in the design of flexible antennas. An improved gain that is the most crucial parameter, especially for 5G applications, can be improved by using metamaterials [54]. AMC, EBG, as well as SRR-based antennas, are reviewed in the article to provide an insight into the MTMs [55,56,57].

Single element antennas are still not suitable for improving the channel capacity. Thus, MIMO antennas are the best choice for improving the channel capacity. Slot-based, DGS- based, Metamaterial-based, DRA-based, and transparent MIMO flexible antennas are reviewed in the article. The orientation of the individual elements with a low mutual coupling is very important in the MIMO implantation. Isolation can be improved by parasitic elements [66], meander lines [74], DGS [77,81,82], etc.

Various studies have been carried out in the field of flexible antennas, especially in the fifth-generation technology. However, here we discuss some of the significant future directions in the flexible antenna design field.

➢ Development of a novel flexible antenna for fifth generation wireless communication which involves the newly released sub-6 GHz bands which contribute to a high data rate, low latency and high-speed communication.
➢ The gain of the flexible antenna is a major design consideration. Therefore, the flexible antenna design with an enhanced gain using various gain enhancement techniques can be conducted.
➢ Flexible devices having less space integrate antenna that are small in volume. To achieve a good bandwidth while keeping the antenna volume low, is a challenge. Therefore, research in such a direction needs further investigation.
➢ Antenna miniaturization using several techniques can be carried out in the specified frequency band since the flexible antennas for the abovesaid band can contribute to different applications, including IoT, mobile communication, and medical applications that need antennas to be highly flexible and compact and contribute to a high-speed communication.
➢ Development of a flexible reconfigurable antenna has a future scope in the design of flexible antennas.
➢ Wireless on-body communication utilize flexible antennas to be integrated onto different parts of the body. Therefore, a wearable antenna for body area networks is a major research area that needs attention.
➢ Flexible implanted antenna designs can be conducted for various medical applications.

## 7. Conclusions

In this article, a detailed review of the flexible antennas for the sub-6 GHz 5G band is discussed, highlighting the choice of the conductive as well as the substrate materials needed for the fabrication of flexible antennas. The choice of the conductive and substrate materials for the flexible antenna fabrication has to be carried out based on the intended applications, capability to integrate over any rigid and non-rigid surfaces, low cost, and ease of fabrication process. The parameters needed for the design and analysis of the flexible antennas are discussed with an emphasis on several antenna parameters such as gain, radiation pattern, bandwidth, efficiency, etc. A bending analysis and a SAR analysis of the antenna and its relevance are discussed in detail concerning many single-elements, array-based, as well as MIMO flexible antennas for sub-6 GHz applications. The specific absorption rate has to be within the permissible level when the antenna is utilized for a wireless body area network (WBAN). The design challenges of the flexible antennas and the best possible solutions are also discussed in this article.

## Figures and Tables

**Table 1:** Sub-6 GHz mid-band spectrum. Reprinted from ref. [7].

Countries	1 to 3 GHz Band	3 to 4 GHz Band	4 to 5 GHz Band
Korea	2.30 to 2.39 GHz	3.40 to 3.70 GHz, 3.70 to 4.00 GHz	
Japan		3.60 to 4.10 GHz	4.50 to 4.90 GHz
China	2.50 or 2.60 GHz (B41 or n41)	3.30 to 3.60 GHz	4.50 to 5.00 GHz
EU		3.40 to 3.80 GHz	
UK		3.40 to 3.80 GHz	
Germany		3.40 to 3.80 GHz	
France		3.46 to 3.80 GHz	
Italy		3.60 to 3.80 GHz	
USA	2.50 or 2.60 GHz	3.45 to 3.70 GHz, 3.70 to 3.98 GHz	4.49 to 4.99 GHz
Canada		3.47 to 3.65 GHz, 3.65 to 4.00 GHz	
Australia		3.40 to 3.70 GHz	
India		3.30 to 3.60 GHz	

**Table 2:** Conductive materials for a flexible antenna. Reprinted from refs. [27,28].

Conductive Materials	Conductivity, σ (S/m)	Advantages	Disadvantages
Silver Nanoparticles	$2.173\times 10^{7}$	High electrical conductivity	Expensive
PANI/CCo Composite	$7.3\times 10^{3}$	Good mechanical properties	Low conductivity
Paper	$4.2\times 10^{5}$	Easy availability	Low Stability
Meshed Fabric	$2\times 10^{5}$	Easy availability, Low profile	Low fabrication accuracy
Nano Flakes	$6\times 10^{5}$	Good thermal stability	Less radiation efficiency
Graphene Assembled Film	$1\times 10^{6}$	High electrical conductivity	Expensive fabrication
Copper Nanoparticle/Copper Mesh	$1\times 10^{6}$	Good conductivity, good radiation characteristics	High oxide formation
AgHT	$2.2\times 10^{5}$	Transparency	Low resistivity
AgNW	$8.1\times 10^{5}$	Conformality	Low durability, high roughness

**Table 3:** Different flexible materials and their properties. Reprinted from ref. [27].

Properties	Polymer	Textile	Paper	Fluidic
Dielectric Loss/Loss Tangent	Low Loss	Low Loss	Medium Loss	High Loss
Tensile Strength	High	Low	Low	Low
Deformability	Low	High	High	High
Thermal Stability	High	Low	Low	Low
Fabrication Complexity	Simple	Complex	Simple	Complex
Robustness to Wetness	High	Medium	Low	Low
Cost of Fabrication	Medium	Low	Low	High
Weight	Medium	Low	Low	Medium
Overall Size	Small	Large	Large	Small

**Table 4:** Comparison of the various substrate materials in flexible antenna design.

Substrate	$\mathrm{Dielectric}\;\mathrm{Constant}\;\varepsilon_{r}$	Dielectric loss (tanδ)	Thickness (mm)	Advantages	Disadvantages
Rogers (RO 3003)	3	0.0013	1.52	Low moisture absorption, good dimensional stability	Low flexibility
PDMS	2.7	0.013	1	Water-resistant, good chemical stability	Fabrication complexity, high cost
Photo Paper	2.85	0.05	0.254	Low profile, easily available	Highly fragile, high moisture absorption
Kapton Polymide	3.5	0.002	0.10	Good electrical stability, Easy to fabricate	High moisture absorption
Tac/Felt	2.2	0.0009	1.52	Low cost, easily available	High moisture absorption, less smooth
LCP	2.9	0.022	0.1	High heat resistance, good mechanical properties	Fabrication complexity, High cost
PEN	2.6	0.005	0.1	Good electrical and mechanical properties	Low thermal stability
PET	3.2	0.022	0.1	Transparency, good thermal and electrical properties, good moisture stability, and conformality	Low thermal stability

**Table 5:** Different tissue layers and their thickness in different parts of the body (Reprinted with permission from ref. [43]. 2018 Ashyap et al.).

Body Parts	Head	Chest	Abdomen	Upper Arm	Forearm	Thigh	Lower Leg
Skin (Thickness in mm)	4	2	2	2	2	2	2
Fat (Thickness in mm)	-	4	7	6.1	4.3	8.8	6.3
Muscle (Thickness in mm)	9.5	38	25	20.3	14.9	28.7	20.8
Bone (Thickness in mm)	20.5	46	-	9.1	6.3	13	9.4
Grey Matter (Thickness in mm)	51	-	-	-	-	-	-
Heart (Thickness in mm)	-	-	56	-	-	-	-

**Table 6:** Dielectric characteristics of tissue layers for different frequencies. Reprinted with permission from ref. [45]. Copyright 2021 ETE Journal of Research.

Tissue Layers	$\mathrm{Dielectric}\;\mathrm{Constant}\;\varepsilon_{r}$	Conductivity σ (S/m)
2.4 GHz		
Skin	42.85	1.59
Fat	10.82	0.27
Muscle	53.6	1.81
3.5 GHz		
Skin	41.41	2.35
Fat	10.5	0.42
Muscle	52.12	2.72
5 GHz		
Skin	35.78	3.06
Fat	5.03	0.24
Muscle	49.54	4.04

**Table 7:** Summary of a single element flexible antenna for a sub-6 GHz 5G band.

Ref	Size ($mm^{3}$)	Bandwidth (GHz)	Gain (dBi)	SAR	Bending/Effect	Substrate, $\varepsilon_{r}$, tan*δ*	Advantages	Disadvantages
[29]	58×78×0.9	3.89–5.9	3	-	x-axis and y-axis/Slight variation in resonant frequency	PET 3.2, 0.022	Transparent	Thickness of substrate
[47]	25×32×0.064	2.85–5.35 2.35–2.5 3.18–3.82 4.15–5.42	3.75 2.1 2.8 3.5	-	Bending across different radii/Negligible effect on return loss	Ultra-thin Rogers, 3.52, 0.003	Compact size	Negative gain values at cut-off frequencies
[48]	50×50×0.088	3.13–4.42	-	-	Tensile and compressive bending across different radii/High durability of antenna even after 100 bending cycles	PET, 3.5, 0.022	Miniatured size	Slight difference in input impedance of the designed and fabricated antenna
[50]	20×32×0.1	2.38–2.79 3.27–4.05 4.80–8.44	0.65 2.26 2.6	0.93 (2.4 GHz) 0.89 (3.5 GHz) 1.92 (5 GHz) (10 g tissue)	E-plane and H-plane bending across different radii/Slight difference in resonance for E-plane bending	LCP 2.9, 0.002	Low antenna volume	Low gain
[51]	70×70×0.11	2.038–3.745	3 (2.45 GHz) 5 (3.42 GHz)	0.908 (forearm) 0.782 (chest) (1 g tissue) 0.468 (forearm) 0.342 (chest) (10 g of tissue)	-	Kapton Polyimide 3.5, 0.002	Good radiation efficiency, highly flexible	Complex structure
[52]	55×40×0.125	1.77–6.95	2.5–5.9	-	Bending across different radii/Stable performance	Kapton Polyimide 3.5, 0.007	Simple configuration	Variation in measured results due to material specifications
[53]	60×60×1.52	2.40–2.54 3.38–3.52	6.7 8.9	0.2 and 0.1 (2.45 GHz) (1 g of tissue) 0.1 and 0.04 (3.45 GHz) For 10 g of tissue	x- and y-axis bending across different radii/A negligible shift in frequency compared to a flat condition	Felt/Tac 2.2, 0.0009	Good gain and efficiency	Less conformal
[55]	30×33×0.1	2.52–2.62 3.31–3.64	2.39 (2.60 GHz) 1.75 (3.48 GHz)	-	Convex bending across different radii/No significant change in antenna performance	Polyimide 3.5, 0.002	Low volume	Radiation pattern not stable
[56]	37×31×0.1	3.4–3.78 5.7–6.9	-	-	Bending across different degrees/30° bending showed triple band variation	LCP 2.9, 0.0025	Conformal due to the low thickness of the substrate	Complex fabrication
[57]	86×86×0.1	3.1–3.9 5.4–6.2	9.078 7.665 (With reflector)	0.0683 (3.5 GHz) 0.333 (5.8 GHz) (1 g of tissue) 0.226 (3.5 GHz) 0.0814 (5.8 GHz) (10 g of tissue)	-	Rogers ULTRALAM 3850 2.9, 0.0025	Improved gain, reduced SAR	Large volume
[60]	14×3.25×3.25	5.4–5.9	4.3 (5.8 GHz)	0.105 (1 g of tissue), 0.27 (10 g of tissue)	-	Fabric	Good gain	Low bandwidth

**Table 8:** Summary of the flexible MIMO antenna for the sub-6 GHz 5G band.

Ref	Size $\left(mm^{3}\right)$	Bandwidth (GHz)	Isolation (dB)	Number of Elements, Isolation Technique	Gain (dB)	SAR	Bending/Effect	ECC	DG (dB)	TARC (dB)	MEG (dB)	CCL (Bits/s/Hz)
[73]	59×29×0.1	2.35–2.55 3.37–3.60 4.92–5.37	19	2, Defected ground plane	3.79	0.64 for 10 g tissue	E- and H-plane bending/Negligible effect	0.05	9.95	−10	-	-
[74]	110×97×1.4	1.5–3.8 4.2–6.2	25–33	4, Meander lines	2.0–5.0	<1.6	-	0.1	>9.8	−10	3	-
[75]	55×35×1	3–11	19	2, Stub at the ground surface	-	0.62 for 1 g of tissue	-	0.06	<9.975	-	<0.2	-
[76]	110×116×1	2.22–3.85	30	4, Stub extended from the ground plane	0.56	-	Horizontal and Vertical Bending/Deterioration in reflection coefficient	0.2	-	-	-	0.2
[77]	55×55×0.2	3.34–5.01 8.9–9.2	20	4, Defected ground Plane	4	-	x-axis and y-axis bending/Negligible effect on bandwidth	0.17	-	-	-	-
[81]	Case 1: 50×35×1.85 Case 2: 70×25×1.85	4.65–4.97 4.67–4.94	17.44 17.47	2, Partial ground plane 2, the distance between the elements	1.83 1.65	-	-	0.02 0.02	9.96 9.94	>−15 >−12	1 1	0.10 0.15
[82]	66×45×0.625	2.21–6	15	4, Defected ground plane	0.53	-	A 300° horizontal and vertical bending/Variation in gain	<0.016	~10	-	-	-

**Table 9:** Differences in the SAR values correspond to the changes in the distance from the human body. Reprinted with permission from ref. [83]. Copyright 2017 Taylor &Francis Ltd.

Scaled Net Input Power 100 mW	Maximum Mean SAR for 1 g Tissue (W/kg)	Maximum Mean SAR for 10 g Tissue (W/kg)
Distance (in mm)		
0.0	0.864	0.273
1.0	0.420	0.199
2.0	0.203	0.104
3.0	0.134	0.077
4.0	0.107	0.070
5.0	0.100	0.069
6.0	0.094	0.068

## Data Availability

Not applicable.
